# Solar photovoltaic wood racking mechanical design for trellis-based agrivoltaics

**DOI:** 10.1371/journal.pone.0294682

**Published:** 2023-12-01

**Authors:** Uzair Jamil, Nicholas Vandewetering, Joshua M. Pearce

**Affiliations:** 1 Department of Mechanical and Materials Engineering, Western University, London, Canada; 2 Department of Civil & Environmental Engineering, Western University, London, Canada; 3 Department of Electrical & Computer Engineering, University of Western Ontario, London, Ontario; 4 Ivey School of Business, University of Western Ontario, London, Ontario; CINVESTAV IPN: Centro de Investigacion y de Estudios Avanzados del Instituto Politecnico Nacional, MEXICO

## Abstract

Using a trellis to plant vegetables and fruits can double or triple the yield per acre as well as reduce diseases/pests, ease harvesting and make cleaner produce. Cultivars such as cucumbers, grapes, kiwi, melons, peas, passion fruit, pole beans, pumpkins, strawberries, squash, and tomatoes are all grown with trellises. Many of these cultivars showed increased yield with partial shading with semi-transparent solar photovoltaic (PV) systems. To further increase the efficiency of trellis-based growing systems, this study investigates novel low-cost, open-source, sustainable, wood-based PV racking designs for agrivoltaic applications. Design calculations are made to ensure these racks exceed Canadian building code standards, which with snow loads surpass those of most of the world. A complete bill of materials, fabrication instructions, and proof-of-concept prototypes are provided for three system topographies (sloped, T-shaped and inverse Y) along with economic analysis. In addition, to being cost competitive, the designs can act as trellis supports and be used for irrigation/fertigation purposes. The results indicate that these racking structures have enormous promise both agriculturally and energetically. If employed on only grape farms inside Canada, 10 GW of PV potential is made available, which is more than twice the total current installed PV in Canada.

## 1. Introduction

Agrivoltaics refers to the dual utilization of land for clean electricity generation through solar photovoltaic (PV) technology and agriculture [1–5]. The technology intends to answer the land-use conflict associated with large-scale PV farms when these PV plants are installed on farmland [6–9]. In agrivoltaics, PV installation is carried out in a manner to have minimal or no adverse impact on agricultural output. This strategy is efficient in minimizing land use competition, increasing land efficiency [10, 11] and enhancing the economic value of farms.

Agrivoltaic systems have proven to be economically favorable as they provide people (especially farmers) with dual stream of revenue–one through generation of electricity and one through the produce/crop yield [4, 12–16]. Despite it being financially feasible, further alleviating the capital costs of the PV system would accelerate its adoption [17–19]. The capital cost of PV racking contributes substantially to the total cost of PV systems [20–22]. These racks costs tend to increase even more when specialty design racks are used for agrivoltaics–such as the stilt mounted configuration with increased racking height instead of between-the-row topologies [23, 24]. An important aspect that needs to be taken care of while developing a low-cost racking design is that it must be sustainable. Responsibly-sourced wood is considered sustainable [25], and it has a negative embodied energy when compared with other PV racking materials [20, 26]. Several low-cost open-source wood racks have been designed previously for fixed-tilt [20], variable-tilt [27] and vertically-mounted PV systems [28]. No open-source wood racks have been developed for trellis-based crops despite the fact that trellises are often constructed with wood [29]. Using trellis to plant vegetables and fruits is an efficient method to grow more crops, twice or thrice the reference amount, in smaller spaces [30–32]. It provides added advantages including reduced diseases and pests, easy harvest as well as cleaner produce [30]. Cultivars such as cucumbers, grapes, kiwi, melons, peas, chayote, nasturtium, loofah, Malabar spinach, passion fruit, pole beans, pumpkins, strawberries, summer squash, and tomatoes are grown using a trellis [30]. These are substantial crops. For example, approximately 7.3 million hectares of agricultural land is dedicated to grape production world-wide [33]. In 2022, global grape production was around 80 million tons [33]. China dominates the grapes market in the world with its 2022 production standing at 15.6 million tons [33]. It was followed by Italy, 8.1 million tons and France, 6.2 million tons [33]. Canada produced approximately 90 thousand tons of grapes in the same year [34]. Niagara Peninsula and Okanagan are the two main grape-growing regions within Canada [35]. Grapes are grown over 32,951 acres of land within the country [36].

This paper proposes novel low-cost, open-source sustainable wood-based PV racking designs for agrivoltaic applications for trellis-based crops. Although other wood-based PV rack designs exist, it is the first wood-based stilt mounted PV structure in the literature. Previous wood-based racking configurations have primarily focused on conventional PV mounting structures. The current study is the first comprehensive evaluation of a PV racking design specifically intended for trellis-based agrivoltaic systems. Calculations are made to make these racks appropriate for Canadian winters and to meet Canadian building code standards. A complete bill of materials (BOM), fabrication instructions, and proof-of-concept prototype are provided along with economic analysis to ascertain the cost for the system and compare the LCOE of it with other racking solutions.

## 2. Literature review

Agrivoltaics is a symbiotic technology that provides several advantages of agriculture or solar farming alone. Agrivoltaics provides electricity through renewable energy means, which reduces carbon footprint as greenhouse gas (GHG) emissions are reduced since solar-based power offsets energy generated through conventional fossil fuel power plants [37]. The reduction in GHG emissions contribute positively to the efforts of climate change as well as to the environment and economy [38]. Several studies have shown that the application of agrivoltaics can enhance crop productivity [39, 40]. Although some shading with PV can be beneficial, excessive shading can indeed reduce plant yields [12]. A study in US indicated that implementation of agrivoltaics on farmland augmented agricultural yields for some crops over 100% (e.g. for peppers) [41], while another study showed 4.9% and 5.6% increase in biomass and fresh weight for sweet corn in Japan [23]. Besides vegetables, investigation into grain crops also showed encouraging results when combined with PV systems [10]. When crop yields increase, it is obvious that land-use efficiency increases for a farm when PV-based electricity is combined with agriculture [10]. The reason for increased agricultural output is that PV arrays create a microclimate beneath the modules that alter air temperature, relative humidity, wind speed and direction as well as soil moisture of the area [42].

The climatic conditions beneath PV are helpful for cultivars as they protect plants from excessive incident irradiation [2, 41, 43]. Moreover, PV panels act as a barrier or shield for crops from other extreme weather conditions such as high winds and hail [2, 41, 43]. In addition, the operating temperature of PV is reduced as crops are grown underneath which in turn increases solar conversion efficiency for PV modules [2, 41, 43]. Overall the global land productivity can increase by 35–73% through adoption of agrivoltaics [11]. Agrivoltaics, when designed appropriately, can minimize agricultural displacement for energy [5, 44]. The technology also conserves water [45–48]. Electricity generated from PVs installed on agricultural land can be used to operate equipment for drip irrigation [49] and vertical growing [50], which use limited amounts of water when compared to water utilization in conventional farming.

In addition to providing advantages such as fresh and local food, agrivoltaics ensures continued agricultural employment, contrary to conventional solar farms which can adversely impact employment related to agriculture [51–53]. The positive influence of agrivoltaics on the health and wellbeing of individuals and the public stems from two mechanisms. Although fresh food on its own benefits human health, agrivoltaics also offsets pollution due to fossil fuels which adversely impacts people’s wellbeing [54]. Thus, the technology improves human health as well as prevents premature deaths by reducing generation of greenhouse gases [55]. Furthermore, the technology helps in decreasing scope 1, 2 and 3 agricultural emissions [56] as electricity from agrivoltaics can be utilized to produce nitrogen fertilizers [57], hydrogen [58–60] or to charge electric vehicles (EV) which can either be used on-farm or off-farm.

Increased electricity output and land-use efficiency due to agrivoltaics provide a financial value and hence increase revenue for a given piece of land [61]. Moreover, installation of PV on farmland can be considered as a hedge against inflation since PV are a capital investment whose value augments with inflation [62].

Agrivoltaics can also be used to power large loads of data centers and computing facilities such as those running Artificial Intelligence servers and cryptocurrency miners [63]. Furthermore, there appears to be an excellent opportunity of using server waste heat for greenhouses, and agrivoltaics to power the servers as well as other loads of the greenhouses [64]. In this regard, semi-transparent PV systems can be used in greenhouse applications which can supplement electricity needs with continued food production [65–67]. Agrivoltaic systems also reduce soil erosion [68], and help generate climates in deserts or barren lands which are helpful for plant growth [68, 69].

Previous studies have indicated immense potential for agrivoltaic deployment on grape farms. A study conducted for India concluded that the economic value of grape agrivoltaics may increase to more than 15 times that of traditional farming [70]. In addition to much higher revenue for famers, 16,000 GWh of electrical energy can be generated if agrivoltaic systems are installed on all vineyards in India, enough to meet the electricity demands of 15 million people [70]. Another investigation in Xinjiang, China, showed minimal or no change in the yield of grapes under agrivoltaics [71]. The land equivalent ratio of integrating PV with grape farms was found to be between 1.27–1.50, thus confirming economic viability of the system [72]. No significant difference in the growth pattern of vines was observed under agrivoltaics [73]. Other than grapes, there are several crops which grow on a trellis such as bitter melons, cucumbers, kiwis, melons and peas [30], which all may be good candidates for the system designed here.

Based on the design, agrivoltaics for grapes can be classified into three types:

1. *“Between the row”* systems in which PV arrays are installed in the space between rows of crops [74]. The height of such arrays does not necessarily have to be increased to be over the height of the trellis.
2. *“PV greenhouses”* in which the transparent roof or other parts of the greenhouse are replaced with PV modules [75].
3. *“Stilt mounted”* systems in which the height of the PV racks is increased and PVs are arranged strategically at intervals to allow a certain amount of sunlight to pass through them [76].

A few companies are offering PV racking designs that are suitable for trellis-based crops or other agrivoltaics where the height of modules must be relatively high. Sun’Agri offers one such design which has shown benefits for crop production [77, 78]. During extreme heat, the vines demonstrated continued growth and required less water [77, 78]. Similarly, Ombrea also designs racking configurations for agrivoltaic applications, especially suitable for crops which require the modules to be placed far from the ground to avoid interference with their growth [79, 80]. Iberdola, a Spanish multinational company, also provides racking solutions for grape farms, which have a positive influence on grape production as it allows for sun light management and temperature [81]. Through the mechanism, arrangement of the modules can be customized to address the requirements of the vineyards. This enables the management of sun exposure and temperature via shading offered by the panels. In China, Huawei provided PV mounting structures for berry plantations in which the solar PV were mounted at a height of approximately 2.9m [82, 83]. ANTAI and Mibet, both Chinese manufacturers, also provide solar racking structures which are suitable for grape farming [84, 85].

## 3. Materials and methods

### 3.1. Selection of material: Wood

Wood is selected as the primary material of choice for the racking structures as it is available in most parts of the world and is a sustainable renewable resource [25]. It also has lower energy needs for processing thus resulting in an overall negative combined embodied energy and carbon when compared with other traditional racking construction materials. Aluminum, one of the most commonly used construction materials for PV racking structures, has more than 5 times embodied CO_2_e/kg of wood [26], giving wood a distinctive edge. Wood has already been shown to be a cost-effective racking material for fixed tilt [20], variable tilt [27], vertical PV [28] and awning designs [86]. Previous work has shown that the economics of wood vs aluminum racks varies widely by geography [87], but it is superior in most of North America.

There is a wide range of wood types available as well as their treatment mechanisms which ensure decay resistance over the lifetime of a PV system. Treated softwood is readily available and is inexpensive in North America, which is considered here. Service life of wood substantially benefits from pressure treatment of wood; thus, the process is widely employed on softwood species. Micronized copper azole, acknowledged for its enhanced safety compared to other methods, thus benefiting humans, animals, and the environment, represents a new generation of wood preservatives [88]. In residential applications, this treatment, commercially available under different names such as MicroPro/LifeWood, Wolmanized Outdoor Wood, Yellawood, and SmartSense, is widely used. One notable advantage of micronized copper azole is its reduced corrosiveness on metal fasteners and compatibility with aluminum, one of the most commonly used materials for solar PV frames. Considering factors such as affordability, abundance, and easy availability as well durability in outdoor conditions, pressure-treated SPF (Spruce, Pine, Fir) lumber was chosen as the preferred material. Depending on weather conditions, pressure-treated lumber can remain intact for up to 40 years without any signs of decay [89]. After the completion of its service life, treated wood can be reused and repurposed to make smaller products such as benches, picture frames etc. or even recycled [90, 91]. Moreover, low-temperature pyrolysis or high temperature gasification has also been found out to be efficient ways of disposing treat wood waste [92].

### 3.2. Dimensional and mechanical characteristics of wooden members

The dimensional and mechanical properties of the wooden members are summarized in Table 1. An important aspect to remember is that the base of any member used for construction should be less than its height so that the member is loaded in its strong axis. Loading the member on its stronger axis results in optimum moment of inertia and first moment of area.

**Table 1:** Properties of wooden members.

Lumber	Lumber Breadth ‘b’ (m)	Lumber Height ‘h’ (m)	Area ‘A’ (m^2^) A = bh	Moment of Inertia ‘I’ (m4) I = bh^3^/12	First Moment of Area ‘Q’ (m3) Q = hA/8
2x4	0.038	0.089	0.003382	2.2324x10^-06^	3.7624x10^-05^
2x6	0.038	0.140	0.005320	8.6893x10^-06^	9.3100 x10^-05^
2x8	0.038	0.184	0.006992	1.9726x10^-05^	1.6081 x10^-04^
2x10	0.038	0.235	0.008930	4.1096x10^-05^	2.6232 x10^-04^
4x10	0.076	0.235	0.017860	8.2193x10^-05^	5.2463 x10^-04^
2x12	0.038	0.286	0.010868	7.4079x10^-05^	3.8853 x10^-04^
4x4	0.089	0.089	0.007921	5.2285x10^-05^	8.8121 x10^-05^
6x6	0.140	0.140	0.019600	3.2013x10^-05^	3.4300 x10^-04^
10x10	0.235	0.235	0.055225	2.5415x10^-04^	1.6222 x10^-03^

### 3.3. T-shaped PV racking design parameters

Four novel T-shaped PV racking designs are proposed with a rated capacity of 920W and 1840W. The design is aimed to be employed for agrivoltaic applications, especially for crops grown using trellises. 460W rated 144HC M6 Bifacial Module was selected to design the geometry of the system [93]. The use of bifacial PV not only enhances electricity production [94, 95], but also aids in snow clearing on the front side [96, 97]. During PV module selection, it is important to ensure that both the rear and front load capacities of the module surpass the calculated design load outlined in Results Section 4.1. The structural capacities of lumber can be found in Section 4.2. The Heliene 144HC M6 Bifacial Modules have a dimension of 2108mm x 1048mm. If modules with different measurements are utilized, the design can be adjusted accordingly to meet the specific module requirements. The racking design proposed has a height of approximately 2m above the ground thus ensuring a 500mm ground clearance, which is enough to ensure snow sliding in even the most extreme northern atmospheres [98]. The structure is designed for Kelowna, Okanagan Valley, British Columbia, with a latitude and longitude of 49.8880° N and 119.4960° W. On average, the winter months typically bring around 25 inches of snowfall per year in Kelowna [99]. There are about 11,086 acres of grape farms inside British Columbia [100]. Of these, 86.8% are in the Okanagan valley [100]. Kelowna consists of 72 vineyards spreading over 800 acres of land [100]. This makes up more than 7% of the total vineyard in BC [100]. Assumptions for the design analysis are detailed in S1 Appendix.

### 3.4. Main design

#### 3.4.1. Two panel design– 920W rated capacity

**Fig 1:**
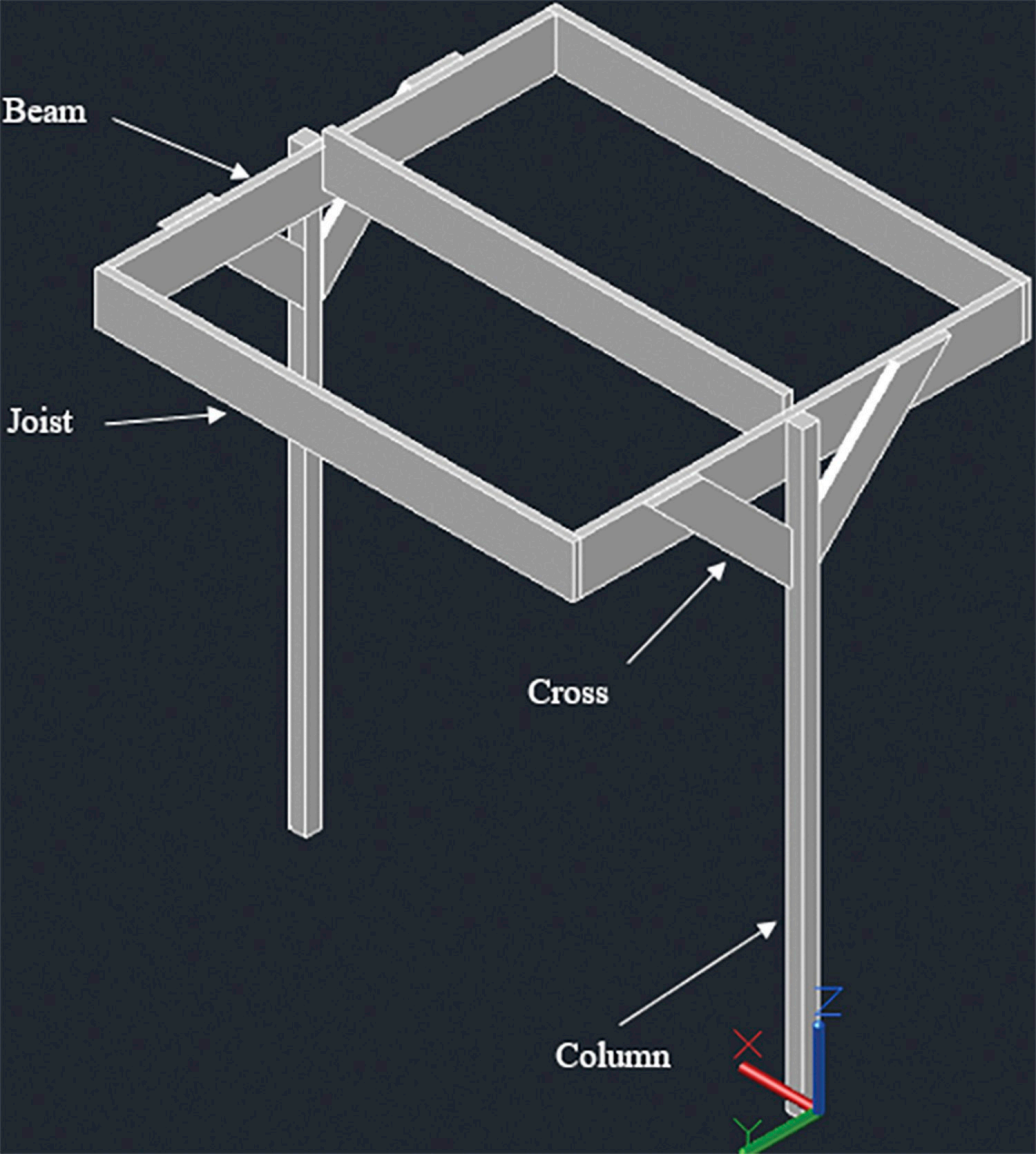
2-panel design of T-shaped wooden racking structure for grape farms.

**Fig 2:**
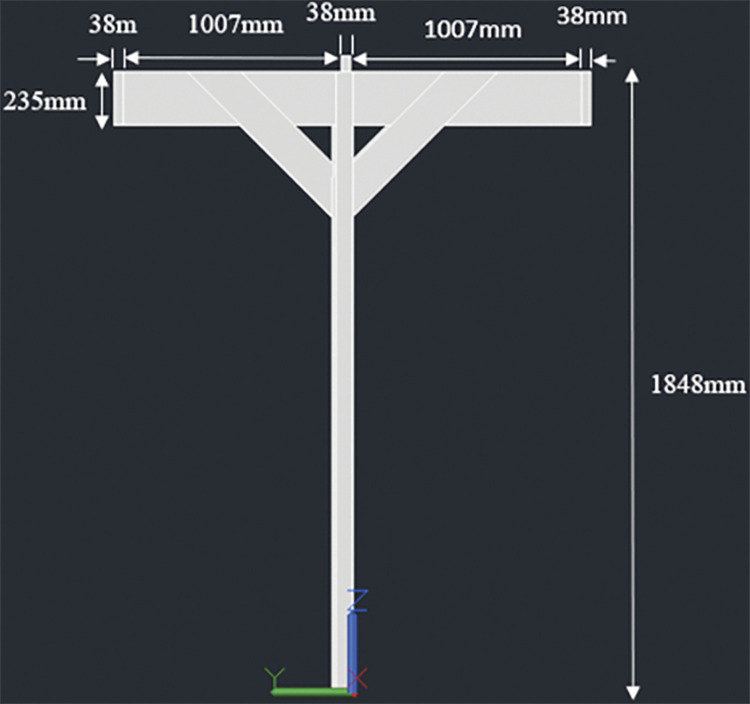
Side view along with dimensions.

**Fig 3:**
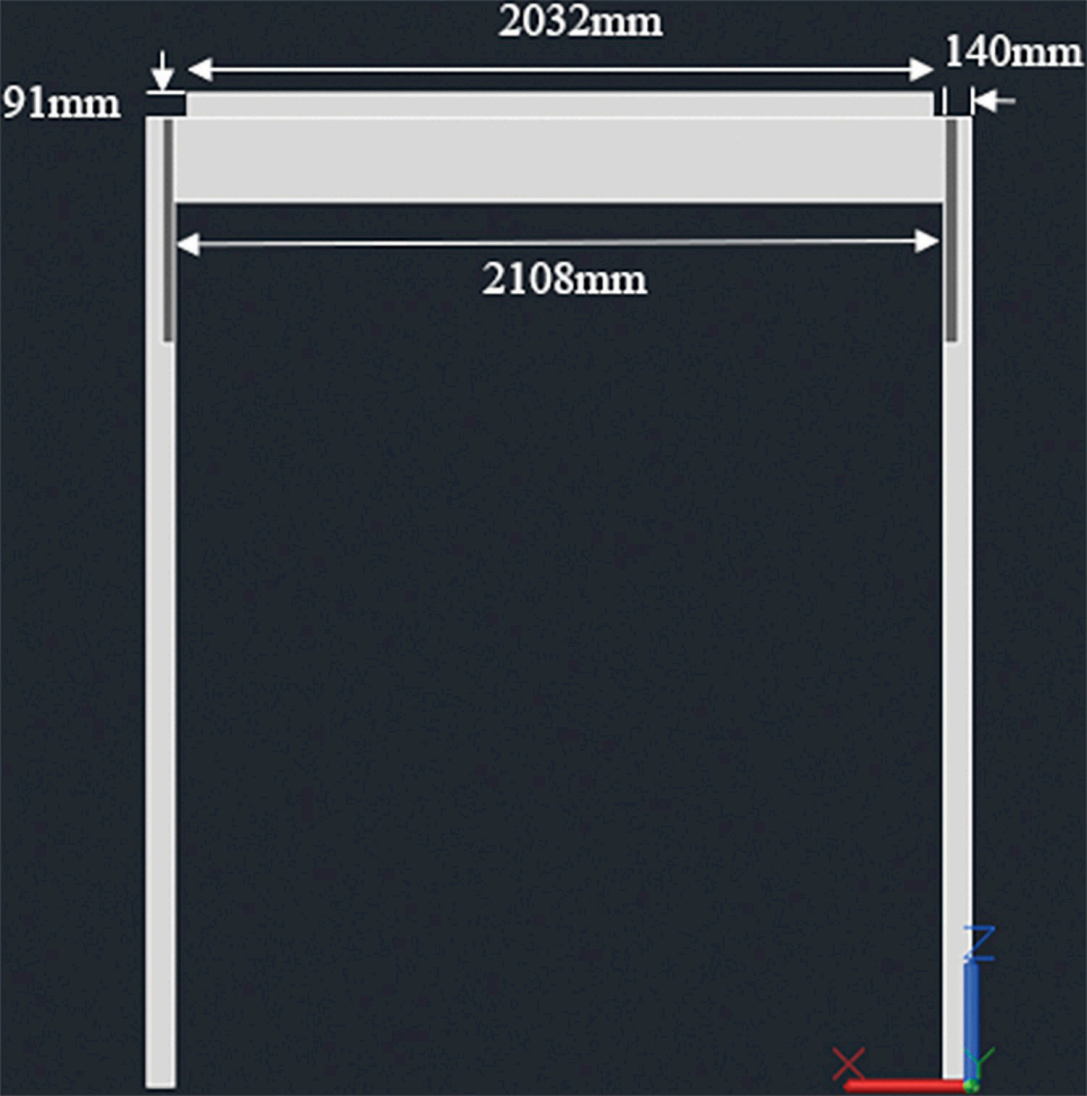
Front view with dimensions.

The racking design consists of two posts, 140mmx140mm in cross section. The height of the posts is kept 1848mm above the ground considering a typical grape trellis height of approximately 1828mm (6 feet). Two beams, 38mm x 235mm in cross section, are attached to these posts (one beam for each post); the connection is made at the midway span of the beams which have a total length of 2052mm. The beams are next connected to three joists, two at both ends and one at the center. The end joists have a length of 2108mm while the center joist has a length of 2032mm. The center joist shares the load of both the panels while the end joists support one panel each. Moreover, the center joist is kept 91mm high to ensure an angle of 5° with the horizontal. The slope will help in removal of debris and other particles from rain over the long use period of the racking structure. Two cross braces are also installed mid-way from the center to the end of end joist for additional support. Figs 1–3 show the assembly of the structure with labelled members and dimensions:

#### 3.4.2. Four panel design– 1840W rated capacity

**Fig 4:**
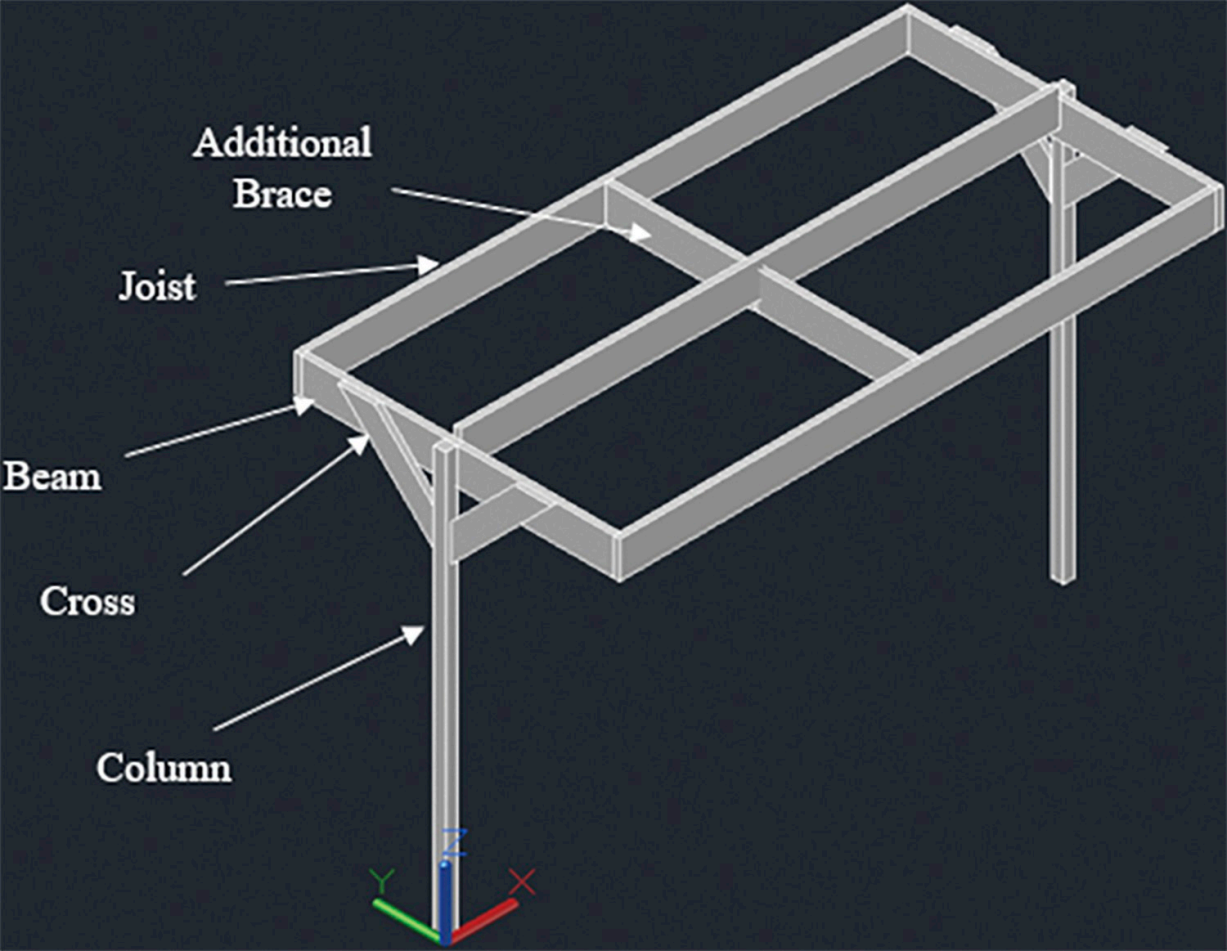
4 panel design for T-shaped racking configuration.

**Fig 5:**
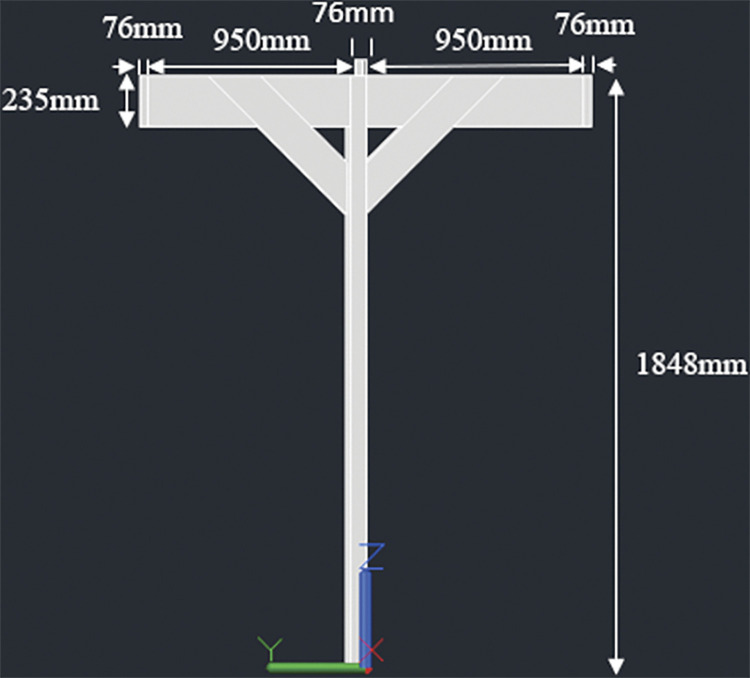
Side view along with dimensions.

**Fig 6:**
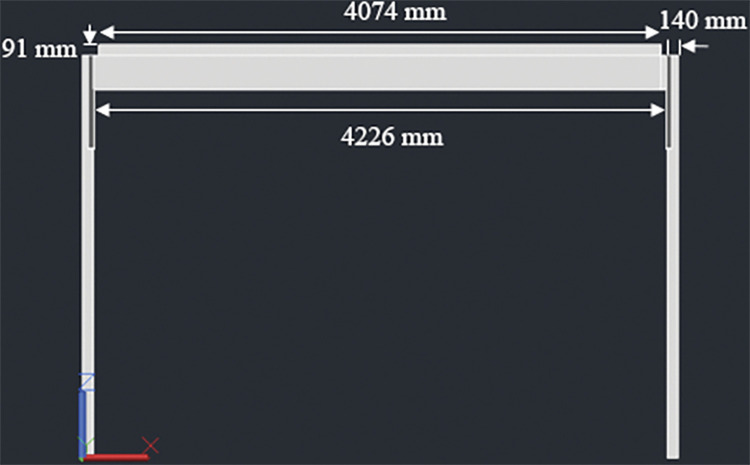
Front view along with dimensions.

The racking design consists of two posts, 140mm x 140mm in cross section. The height of the posts is kept 1848mm above the ground considering a typical grape trellis height of approximately 1828mm (6 feet). Beams, Joists and Cross Braces are 4 inches (76mm) thick–these are made by connecting two members of 2inch (38mmm) thickness. Two beams, 76mm x 235mm in cross section, are attached to these posts (one beam for each post); the connection is made at the midway span of the beams which have a total length of 2052mm. The beams are next connected to three joists, similar to design 1, two at both ends and one at the center. The end joists have a length of 4226mm while the center joist has a length of 4074mm. The center joist shares the load of all the four panels while the end joists support two panels each. As for the 2-panel design, the center joist is kept 91mm high to ensure an angle of 5° with the horizontal. In addition to the cross braces installed similar to design 1, additional bracing is done parallel to the beams at the mid-way span of the joists. Figs 4–6 show the assembly of the structure with labelled members and dimensions:

#### 3.4.3. Sloped T-shaped PV racking design (920W)

**Fig 7:**
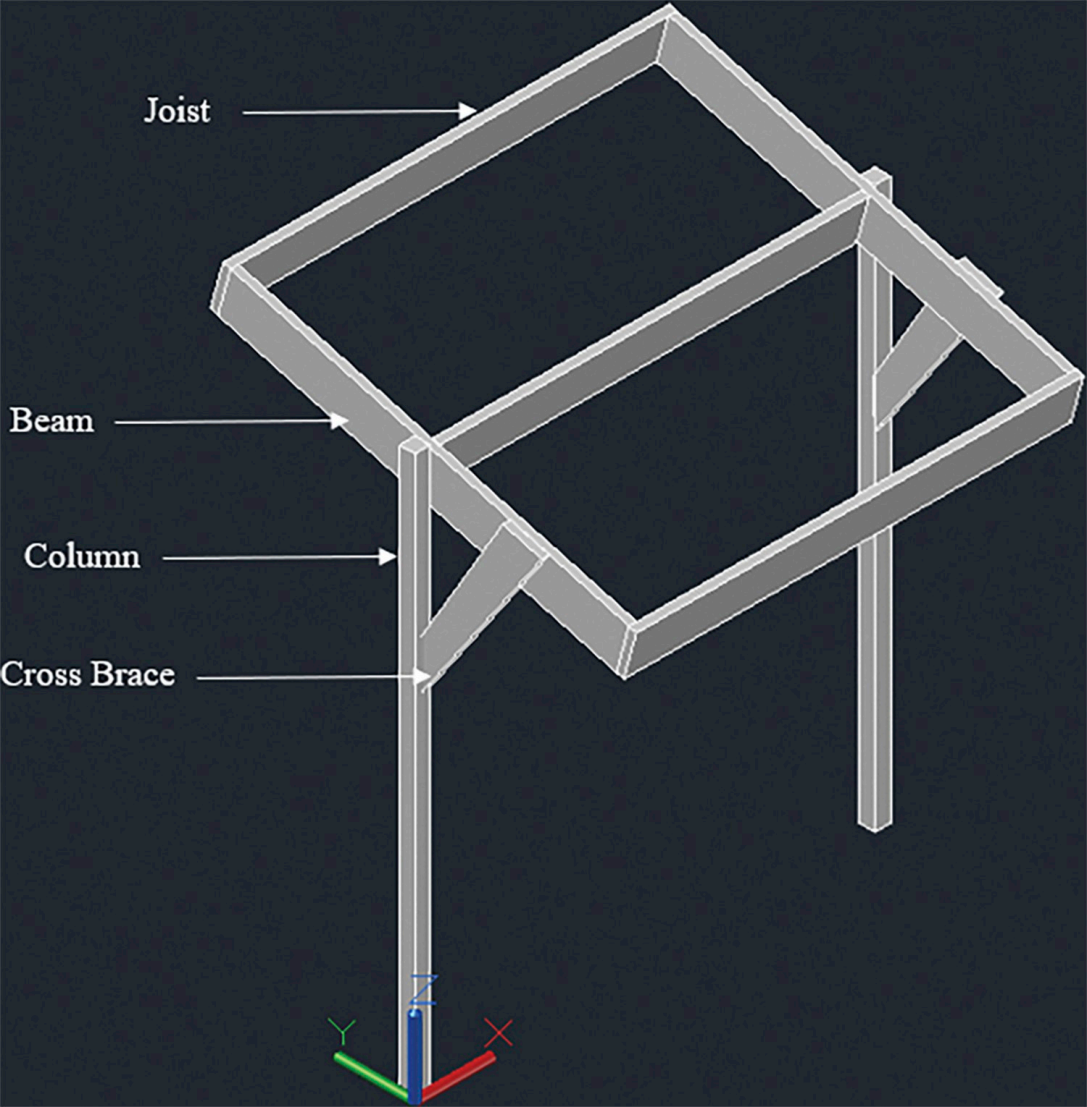
Sloped T-shaped racking configuration.

A variation of the 2-panel racking system is designed to optimize the tilt angle of the PV. The structure remains the same, except that there is one cross brace instead of two for one column. Moreover, there is no need to offset the mid-joist 91mm to give the panels the tilt, as the complete structure is now inclined. The members used to design the system remain the same as for the 920W racking configuration. In addition, the dimensions of the structural members are identical as well. The structural analysis also remains the same, however, there is variation in the truss analysis which is discussed in the Results section. Fig 7 shows the 3-D design of the sloped racking configuration:

#### 3.4.4. Inverse Y racking configuration

**Fig 8:**
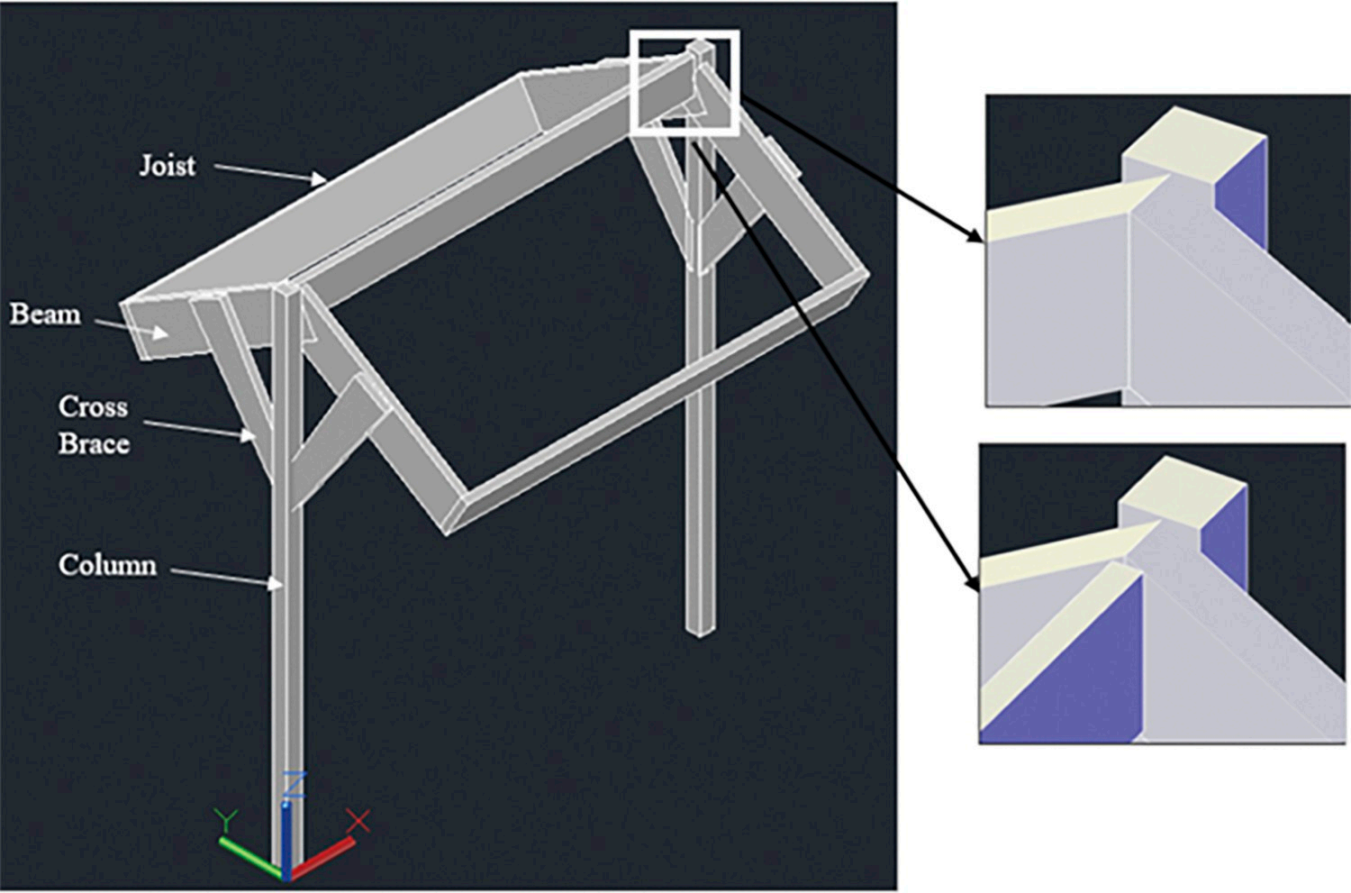
Inverse Y racking configuration.

A variation of the 2-panel racking system is the inverse Y racking configuration. The structure holding the panels remains the same, except that the tilt from the horizontal is higher than 5°. The largest tilt that the structure can sustain is discussed in the Results section. There are two cross braces for one column in this design while there is no requirement to offset mid-joist as the joist and beams holding the modules are themselves inclined. The members used to design the system are the same as for the 920W T-shaped racking configuration. In addition, the dimensions of the structural members are similar as well. The structural analysis remains the same for inverse Y design, however, there is variation in the truss analysis which is discussed in Results section. Fig 8 shows the 3-D concept of the inverse Y racking configuration:

The bill of materials (BOM) for the T-shaped designs is shown in Tables 2 and 3.

**Table 2:** BOM of 2-panels solar photovoltaic wood racking mechanical design for trellis-based agrivoltaics.

			T-Shaped		Sloped			Inverse-Y	
Member Name	Member Dimensions (inches/feet)	Cost per Member ($CAD)	Qty	Price ($CAD)	Qty		Price ($CAD)	Qty	Price ($CAD)
Joists	2x10x8	24.92	3	74.76	3		74.76	3	74.76
Beams	2x10x8	24.92	2	49.84	2		49.84	2	49.84
Cross Braces	2x10x8	24.92	2	49.84	1		24.92	2	49.84
Posts	6x6x10	50.82	2	101.64	2		101.64	2	101.64
Front and End Joists to Beam Connection	2x4 Fence Bracket	0.43	4	1.72	4		1.72	4	1.72
Cross Braces to Beam Connection	2x4 Fence Bracket	0.43	4	1.72	4		1.72	4	1.72
Middle Joist to Beam Connection	2x6 Face-mounted Joist Hanger	1.82	2	3.64	2		3.64	2	3.64
Beam to Post Connection	1/2 Carriage bolt (8"), Nut, and Washer	7.29	4	29.16	4		29.16	8	58.32
Connections	2-1/2 Brown Deck Screws	14.48	1	14.48	1		14.48	1	14.48
Connections	1-1/2 Joist Hanger Nails	5.02	1	5.02	1		5.02	1	5.02
Module to Joists Connections	1/4 Lag Bolt (5")	0.55	6	3.30	6		3.30	6	3.30
Module to Joists Connections	1/4 Square Washer	3.72	6	22.32	6		22.32	6	22.32
3D Printed Spacers	1/2x1	2.00	6	12.00	6		12.00	6	12.00
Concrete for Posts	30 MPa Quirkete concrete	6.98	4	27.92	4		27.92	4	27.92
**Total Cost**				**397.36**		**372.44**			**426.52**
**Total Cost ($/W)**				**0.43**		**0.40**			**0.46**

**Table 3:** BOM of 4-panels solar photovoltaic wood racking mechanical design for trellis-based agrivoltaics.

Member Name	Member Dimensions (inches/feet)	Cost per Member	Quantity	Price (CAD)
Joists	4x12x16	71.36	6	428.16
Beams	4x12x8	35.68	4	142.72
Cross Braces	4x12x8	35.68	4	142.72
Posts	6x6x10	50.82	2	101.64
Additional Braces	2x10x8	24.92	1	24.92
Front and End Joists to Beam Connection	4x4 Joist Hanger	3.88	4	15.52
Cross Braces to Beam Connection	4x4 Joist Hanger	3.88	4	15.52
Middle Joist to Beam Connection	4x6 Face-mounted Joist Hanger	14.98	2	29.96
Beam to Post Connection	1/2 Carriage bolt (8"), Nut, and Washer	7.29	4	29.16
Connections	2-1/2 Brown Deck Screws	14.48	1	14.48
Connections	1-1/2 Joist Hanger Nails	5.02	1	5.02
Module to Joists Connections	1/4 Lag Bolt (5")	0.55	14	7.71
Module to Joists Connections	1/4 Square Washer	3.72	14	52.08
3D Printed Spacers	1/2x1	2.00	14	28.00
Beam to Beam and Joist to Joist connections	30 MPa Quirkete concrete	6.98	4	27.92
Additional Braces to Joist	1/2 Carriage bolt (8"), Nut, and Washer	7.29	12	87.48
Concrete for Posts	2x4 Fence Bracket	0.43	4	1.72
**Total Cost**				**1154.73**
**Total Cost ($/W)**				**0.63**

The following points need to be considered for the materials used in construction of the racking structure:

- The wood used shall be pressure treated
- The fasteners used shall be hot-dip galvanized or zinc plated

To validate the design of the wooden PV racking, a prototype was built. The aim was to ensure that the design was feasible and, if there were any practical issues associated with the assembly of the structure.

### 3.5. Load calculations

**Fig 9:**
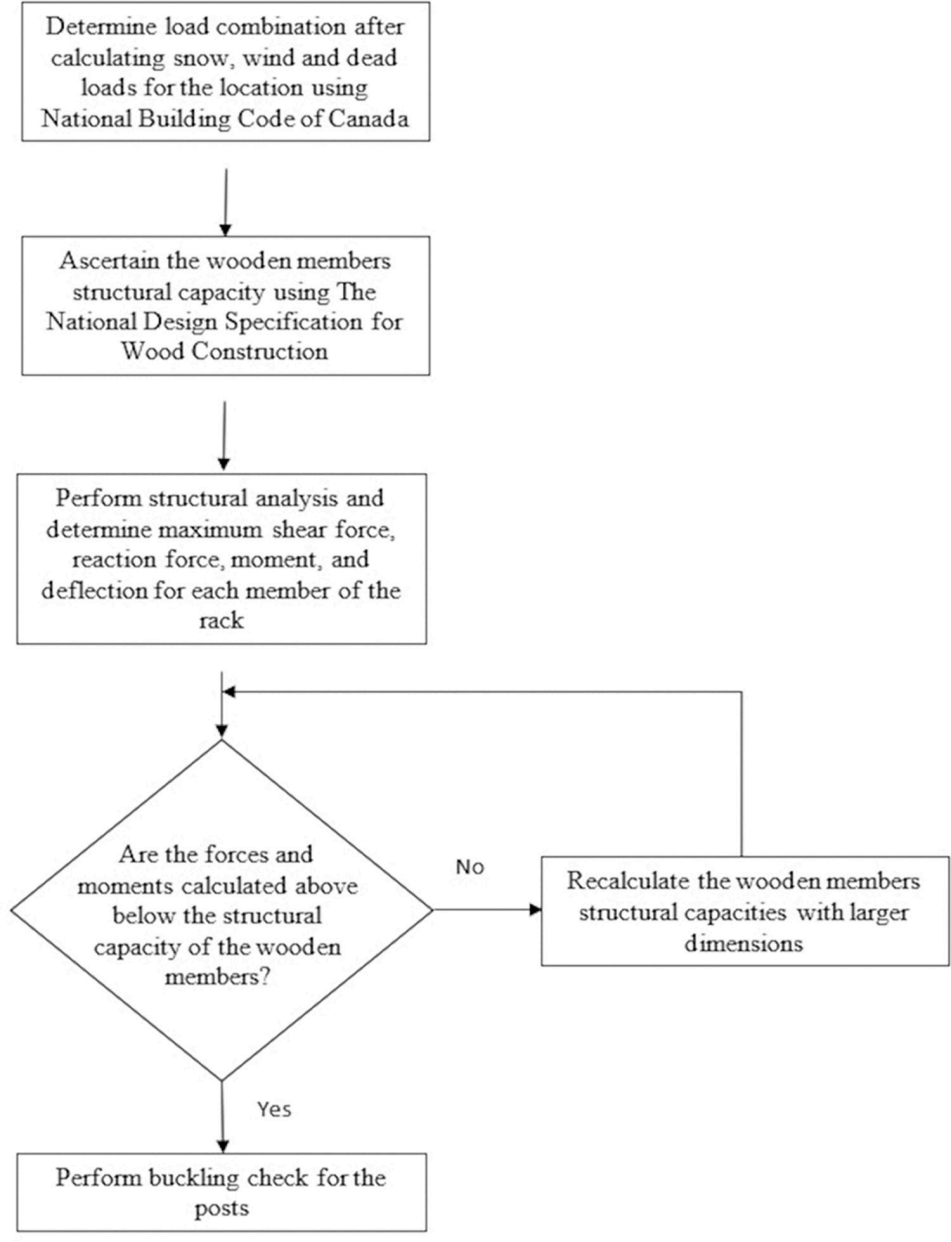
Flowchart describing the design process for wooden racks.

The load calculations are shown in detail in the S2 Appendix. The flow chart shown in Fig 9 summarizes the design steps.

### 3.6. PV System simulations

Energy analysis was performed using the open source System Advisor Model (SAM) [101] for T-shaped racking installed in Kelowna, BC (49.89N°, 119.49°W). Heliene144-HC 460W Bifacial modules were selected for running the analysis [93]. A tilt of 5° was considered for simulation runs. Next, a sensitivity run was performed to determine the energy potential for sloped and inverse-Y PV configuration. Analysis was performed for tilt angles between 5 to 30 degrees (which is the highest tilt that can be achieved with the inverse Y design–details are discussed in Results section).

Simulations were run for the systems with the prescribed number of identical modules. The input parameters for SAM are summarized in S3 Appendix.

## 4. Results

Following results were ascertained for the design of the novel PV racking structure:

### 4.1. Loads

#### 4.1.1. Snow loads

The snow load comes out to be 0.805 kPa using equation B.1-1. As per NBC, however, the minimum specified snow load shall not be less than 1.00 kPa, which is thus considered here.

#### 4.1.2. Wind loads

Following equations B.2-1, B.2-2 and B.2-3, the external pressure, internal pressure and total wind load comes out to be -0.63 kPa, -0.68 kPa and -1.32 kPa respectively.

#### 4.1.3. Dead loads

CanmetENERGY research center at Natural Resources Canada [102] states that the dead load of PV systems, also referred to as the superimposed dead load, should be considered as 0.24 kPa. The weight of the wooden member depends on the member’s dimensions and its capacity to support the load. The weight of wood varies due to changes in moisture content and the presence of knots. For analysis purposes, it is recommended to use the lumber weights provided by the supplier and convert the given weight into a uniformly distributed load in kN/m.

#### 4.1.4. Load combinations

The load combinations that yield the highest positive and negative values can be found in Table 4.

**Table 4:** Load combinations.

Load Combination	Load [kPa]
0.9D + 1.4W -0.5S	2.12
1.25D + 1.5S – 0.4W	2.32

It is important to acknowledge that the load path remains consistent for both the positive and negative cases, with the only distinction being the load direction. Since all connections have the capacity to withstand loads in both directions, and all members possess identical material properties in both directions, the analysis for the negative case is equivalent to the positive case. Therefore, there is no need to consider the negative case, and the analysis will focus solely on the positive case which has a higher load value.

### 4.2. Wooden members structural capacity

In Canada, most of the pressure treated wood is made up of Spruce Pine Fir grades 1 and 2, the mechanical properties [103] of which are given in Table 5.

**Table 5:** Unfactored properties of Spruce Pin Fir wood grades 1 & 2.

Factor	Value (MPa)
f_b_	6.03
f_v_	0.93
f_t_	3.10
f_c_	7.93
E	9652.66
E_min_	3516.33

A summary of resistance factors is given in Table 6. Details are provided in S2 Appendix.

**Table 6:** Resistance factors.

Factor	Value (920W)	Value (1840W)
C_D_	1.15	1.15
C_T_	1.00	1.00
C_M_	1.00, 0.97 and 0.90	1.00, 0.97 and 0.90
C_L_	0.76	0.97
C_fu_	1.2	1.1
C_i_	0.8 and 0.95	0.8 and 0.95
C_r_	1.00	1.00
C_F_	1.10 and 1.00	1.10 and 1.00
C_P_	0.47	0.47

Using equations (B.5-1 – B.5-10) in S2 Appendix, the factored properties of Spruce Pine Fir lumber were calculated which are given in Table 7.

**Table 7:** Factored mechanical properties of Spruce Pine Fir wood.

Factored Capacities	Value 2-panel Design (MPa)	Value 4-panel Design (MPa)
f_b_*	5.55	6.54
f_v_*	0.83	0.83
f_t_	3.14	2.85
f_c_*	2.73	2.73
E*	8253.03	8253.03
Emin*	3006.46	3006.46

Using the factored capacities calculated above as well as the dimensional properties of the wooden members, the resistance values were finally calculated. Table 8 summarizes the resisting capacities for wooden lumber taking the conservative (smaller) numbers that come from the factors ascertained from 2-panel and 4-panel configurations.

**Table 8:** Resisting bending moment, shear force, tensile force and compressive force for different members of Spruce Pine Fir wood.

Lumber	Resisting Bending Moment ‘Mr’ (kN-m)	Resisting Shear Force ‘Vr’ (kN)	Resisting Tensile Force ‘Tr’ (kN)	Resisting Compressive Force ‘Cr’ (kN)
2x4	0.28	1.87	9.65	9.25
2x6	0.68	2.95	15.19	14.55
2x8	1.18	3.87	19.96	19.12
2x10	1.92	4.94	25.49	24.42
4x10	5.03	9.89	50.98	48.83
2x12	2.85	6.02	31.02	29.71
4x4	0.64	4.39	22.61	21.66
6x6	3.28	10.85	55.95	53.59

Once the design load and material properties are known, a structural analysis can be performed to determine the optimal dimensions of lumber required to construct a functional system.

### 4.3. Structural analysis for T-shaped racking

#### 4.3.1. Mid joist

For the middle joist, the tributary width is 1.068m.

Total uniformly distributed load (UDL) comes out to be 2.56 kN/m for 2-panel design using equation (B.6-1). The load comes out to be 2.71kN/m for 4-panel design.

**Fig 10:**
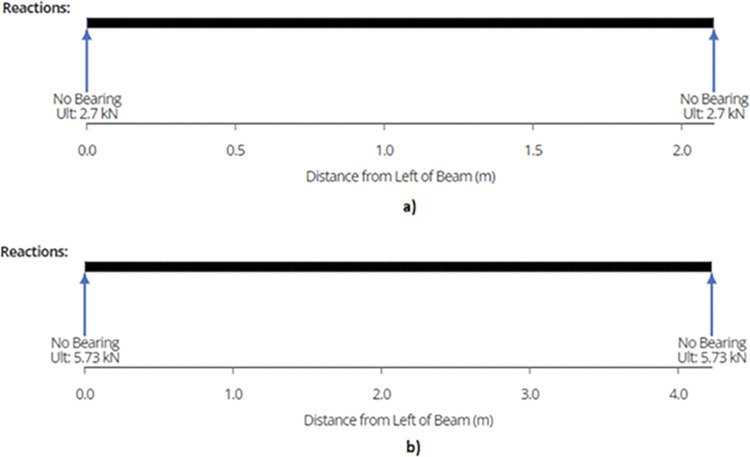
Reactions on a) 2-panel Design and b) 4-panel Design.

**Fig 11:**
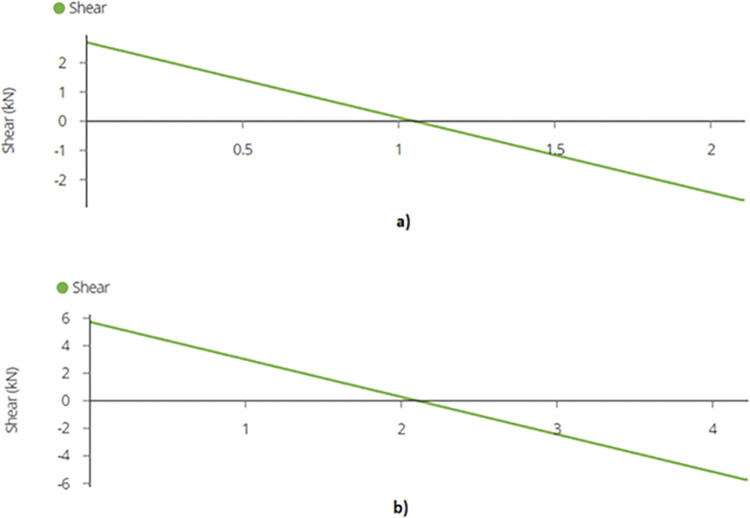
Maximum Shear Force on Mid Joist for a) 2-panel Design and for b) 4-panel Design.

Using equation (B.6-2), maximum shear force or reaction is found out to be 2.70 kN and 5.73 kN for the two designs which occurs at the supports as depicted in Fig 10A and 10B. Shear forces are shown in Fig 11A and 11B. Since the joist is supported at the end by beams, the reaction forces will be transferred to the beams. These reactions will subsequently be used for truss analysis of the beam members.

**Fig 12:**
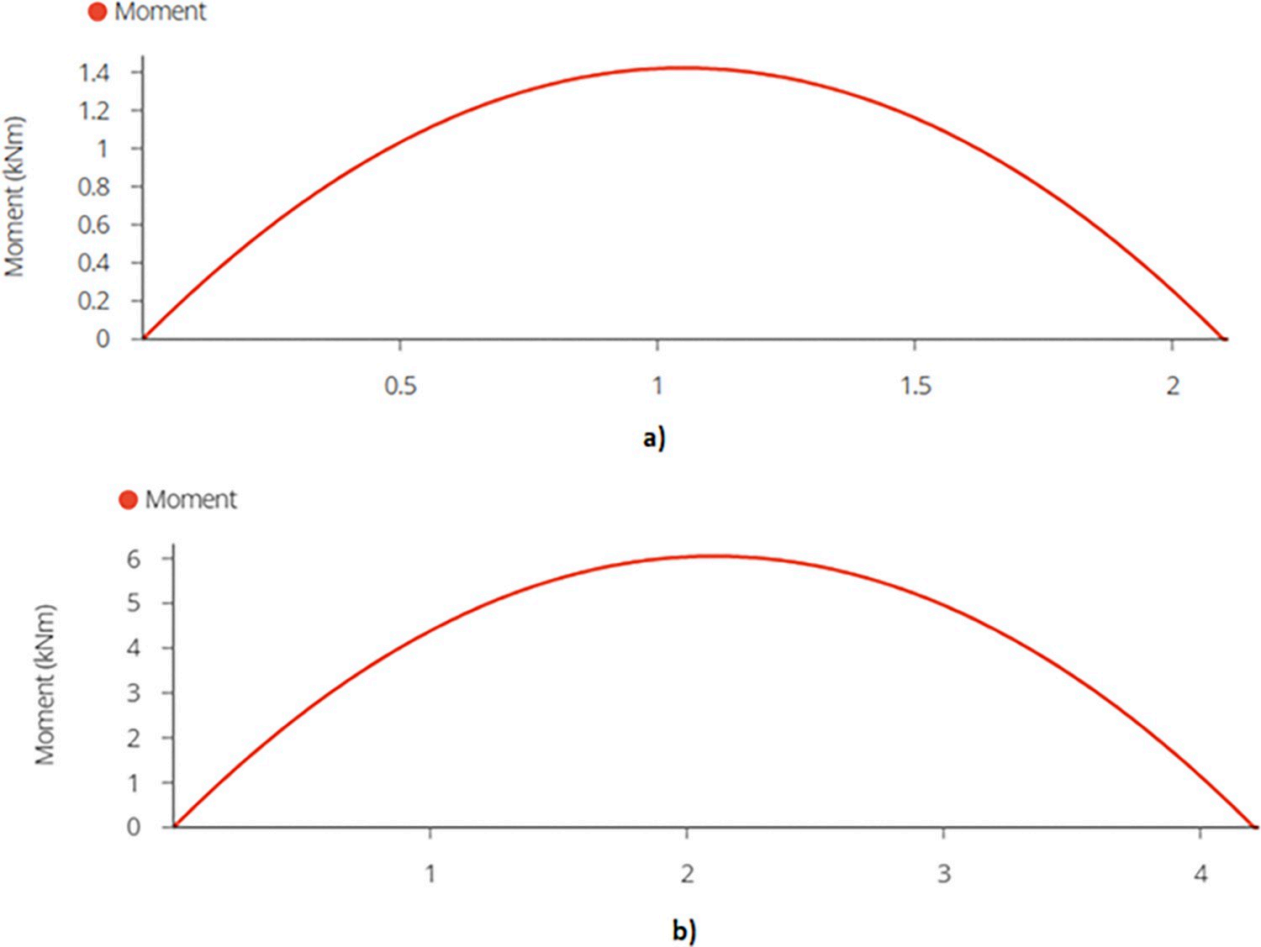
Maximum Bending Moment on Mid Joist for a) 2-panel Design and b) 4-panel Design.

The calculated bending moment comes out to be 1.42 kN-m and 6.04 kN-m for 920W and 1840W racking designs using equation (B.6-3), respectively, which occurs at the mid span of the mid joist. Fig 12A and 12B demonstrate the bending moment diagrams for the two types of racking structures.

**Fig 13:**
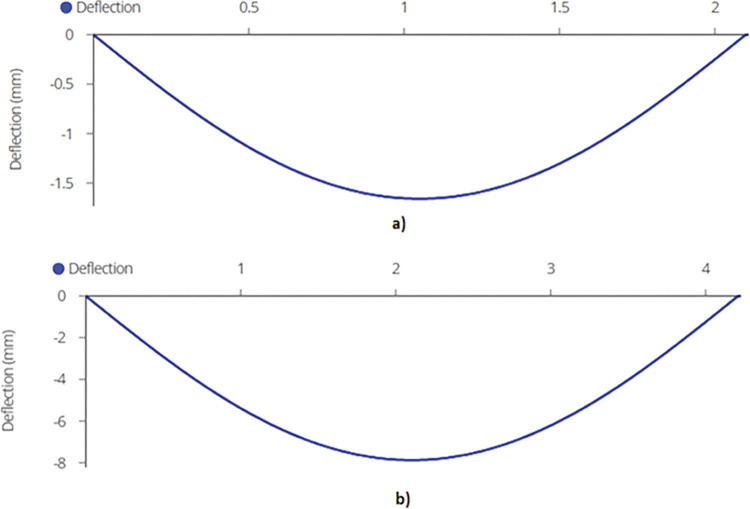
Maximum Deflection on Mid Joist for a) 2-panel Structure and b) 4-panel Structure.

Finally, maximum deflection, which occurs at the mid span of the mid joist, is found out to be 1.66mm and 7.86mm for 2-panel and 4-panel racking configuration using equation (B.6-4) (Fig 13A and 13B).

#### 4.3.2. End joist

For the end joist, the tributary width is 0.534m.

Total uniformly distributed load (UDL) comes out to be 1.32 kN/m and 1.47 kN/m for the two designs from equation (6–1).

The maximum shear force or the reaction for the middle joist is ascertained using equation (B.6-2).

**Fig 14:**
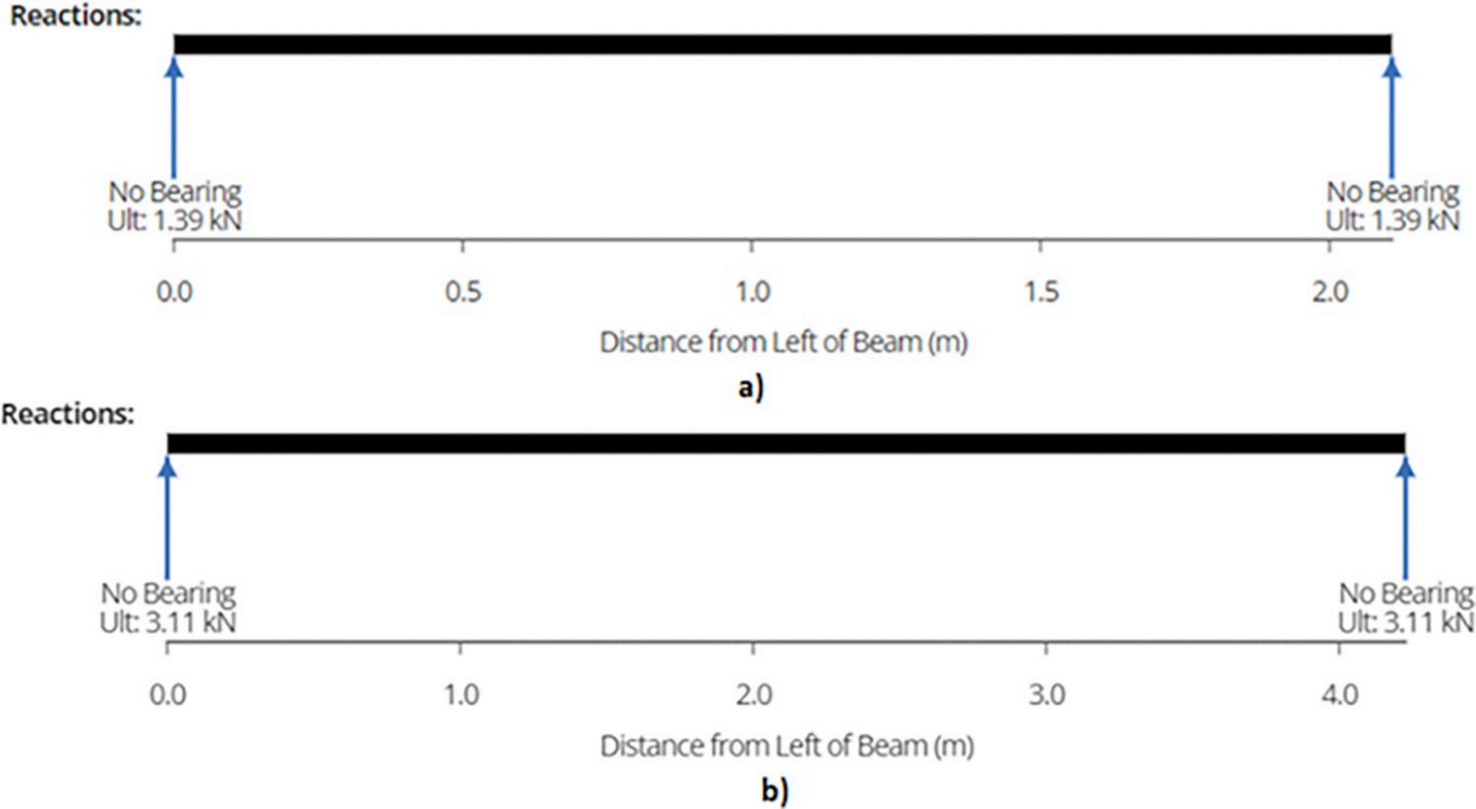
a) Reaction Forces on End Joist for 2-panel Structure and b) Reaction Forces on End Joist for 4-panel Structure.

**Fig 15:**
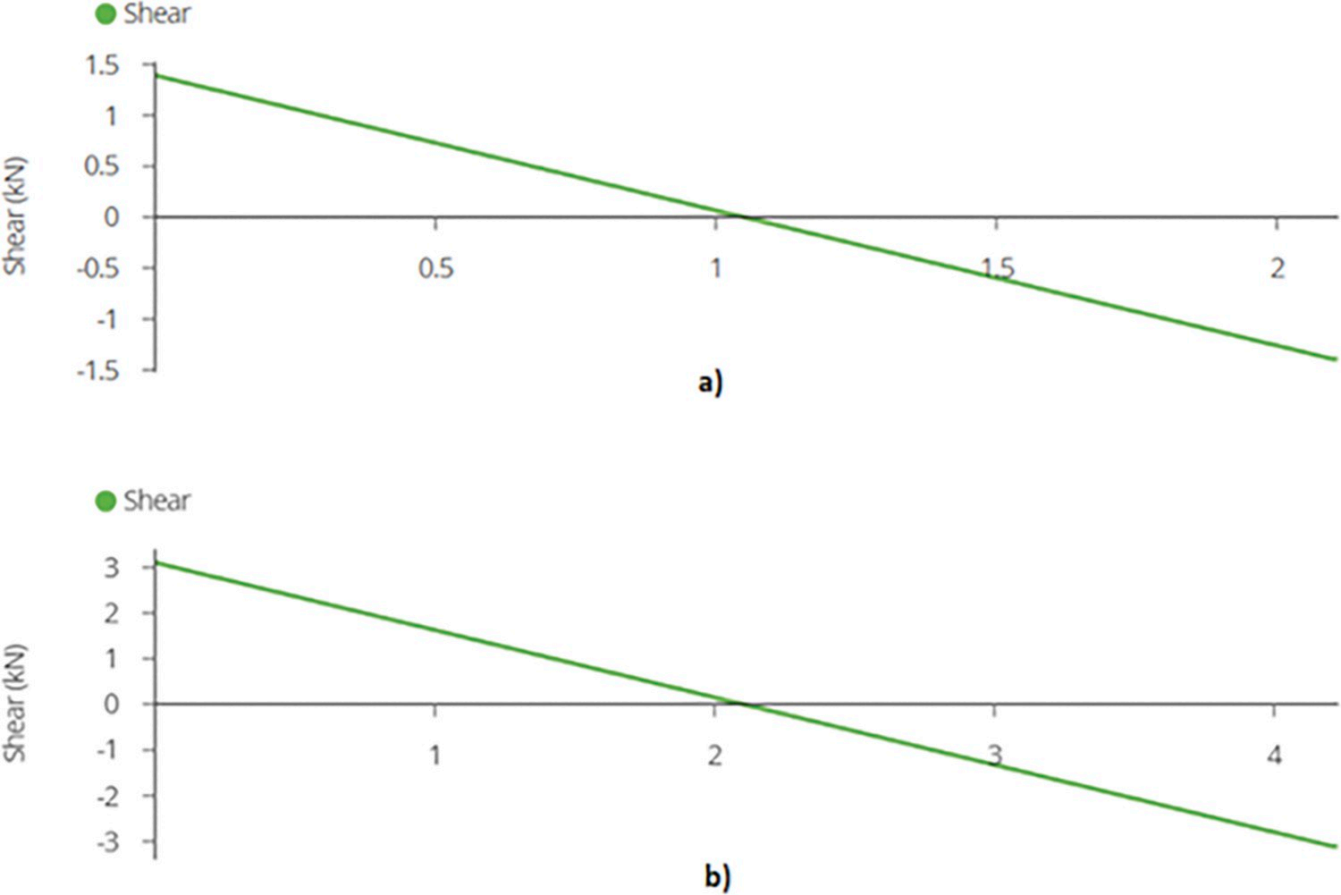
a) Maximum Shear Force on Mid Joist for 920W Racking Design and b) Maximum Shear Force on Mid Joist for 1840W Racking Design.

Maximum shear force or reaction is found out to be 1.39 kN and 3.11 kN for 920W and 1840W racking configurations. Fig 14A and 14B show the reactions while Fig 15A and 15B represent the maximum shear force for the members in each design.

The maximum bending moment is determined using equation (B.6-3).

**Fig 16:**
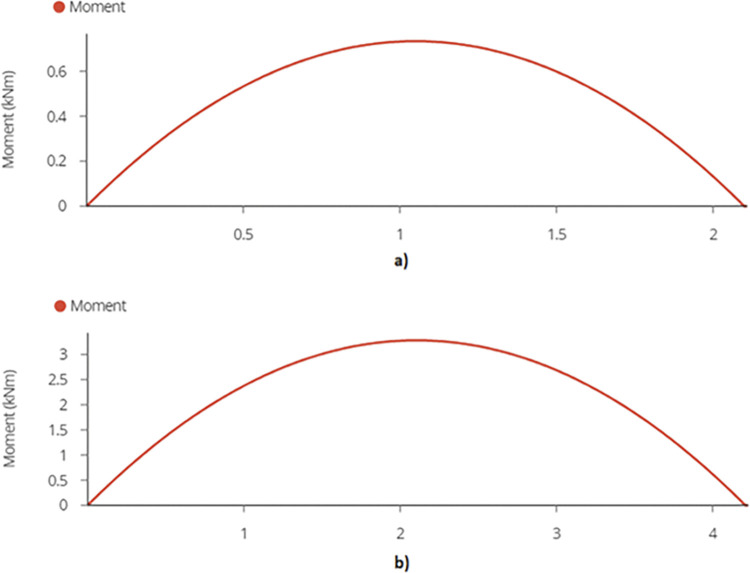
a) Maximum Bending Moment on End Joist– 2-panel Design and b) Maximum Bending Moment on End Joist– 4-panel Design.

The calculated bending moment comes out to be 0.74 kN-m and 3.28 kN-m for 2-panel and 4-panel structures as shown in Fig 16A and 16B.

**Fig 17:**
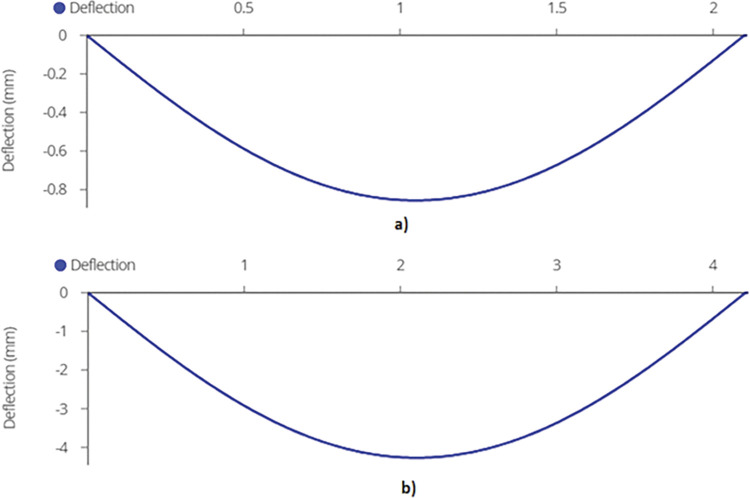
Maximum Deflection on End Joist a) 920W racking configuration and b) 1840W racking configuration.

Finally, maximum deflection is found out to be 1.3mm and 4.27mm using equation (B.6-4). Maximum deflection can be seen in Fig 17A and 17B.

#### 4.3.3. Beams

The reactions from the mid joist and end joists are transferred to the beam and act as point loads. In addition, the own weight of the beam acts as UDL. The beams are supported at the mid span through the 6x6 columns. Also, there are cross braces installed at the mid span from the center of the beam to the end of the beam. These cross braces are at an angle and provide additional support at both ends of the beams. Clearcalcs [104] is used to perform the analysis on the beams.

Three pinned supports are considered in total for the beam:

1. Column support: One at the center 1064mm away from the front or end joist and
2. Cross braces: One 532mm away from the front joist and second 1596mm away from the front joist

The weight of the beam makes up the distributed load and is found out to be 0.085 and 0.12 kN/m for the two designs.

**Fig 18:**
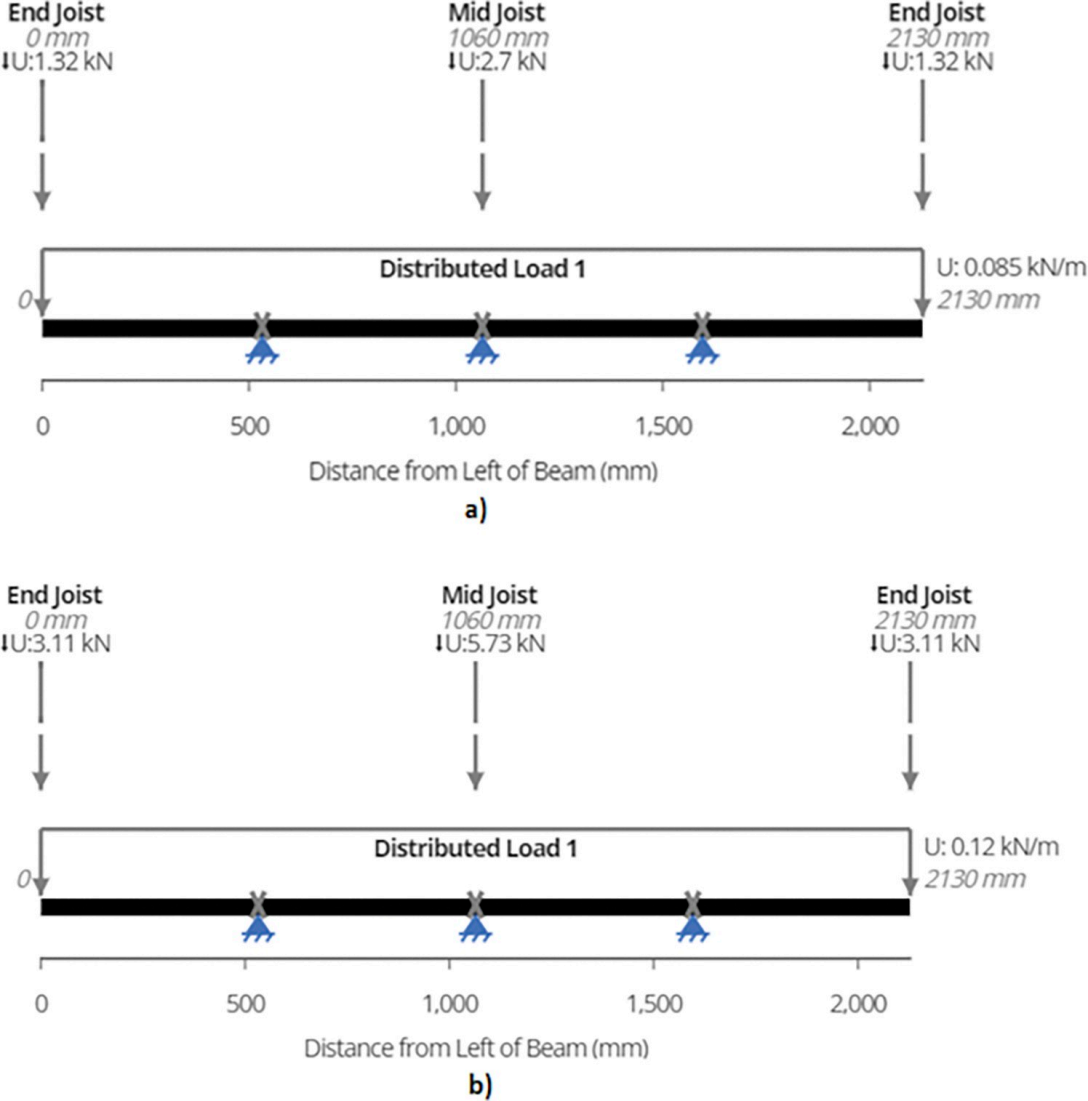
Load distribution as well as the position of Supports for Beam Analysis a) 4-panel Design and b) 2-panel Design.

Three-point loads act on the beam–two from front and end joists while one from the middle joist. For the 2-panel design, the end joists have a reaction of 1.32 kN which acts on either end of the beam while the middle joist has a reaction of 2.70 kN which acts the center of the beam. The values for 4-panel design are considered when analyzing the beams of the that structure. Fig 18A and 18B summarize the loads acting on the beams as well as show where the supports are located.

**Fig 19:**
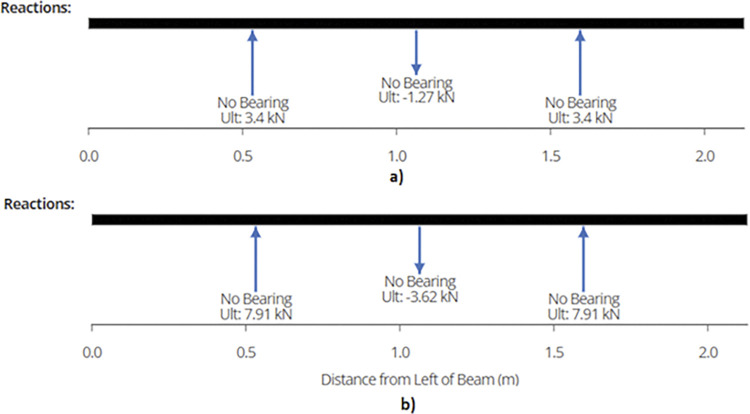
a) Reaction on 920W Structure Beam Supports and b) Reaction on 1840W Structure Beam Supports.

The reactions on the three supports are shown in Fig 19A and 19B.

**Fig 20:**
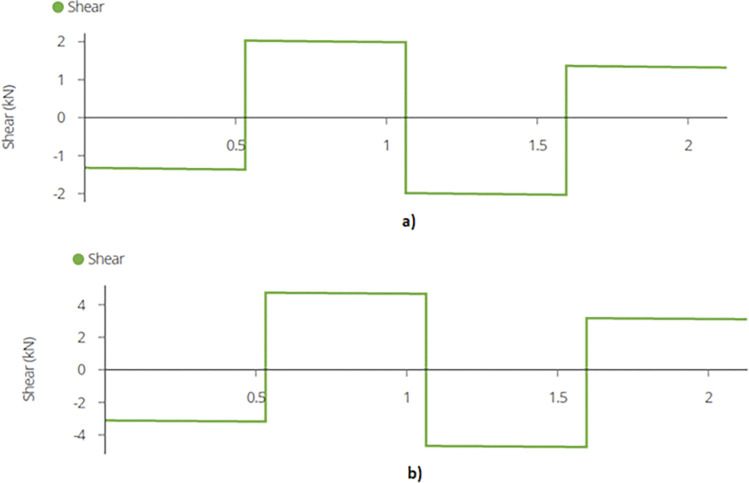
Shear Force Diagram for Beams for a) 2-panel Design and b) 4-panel Design.

The maximum shear force is found out to be 2.03 kN and 4.74kN. Fig 20A and 20B represent the shear force diagram for the beams.

**Fig 21:**
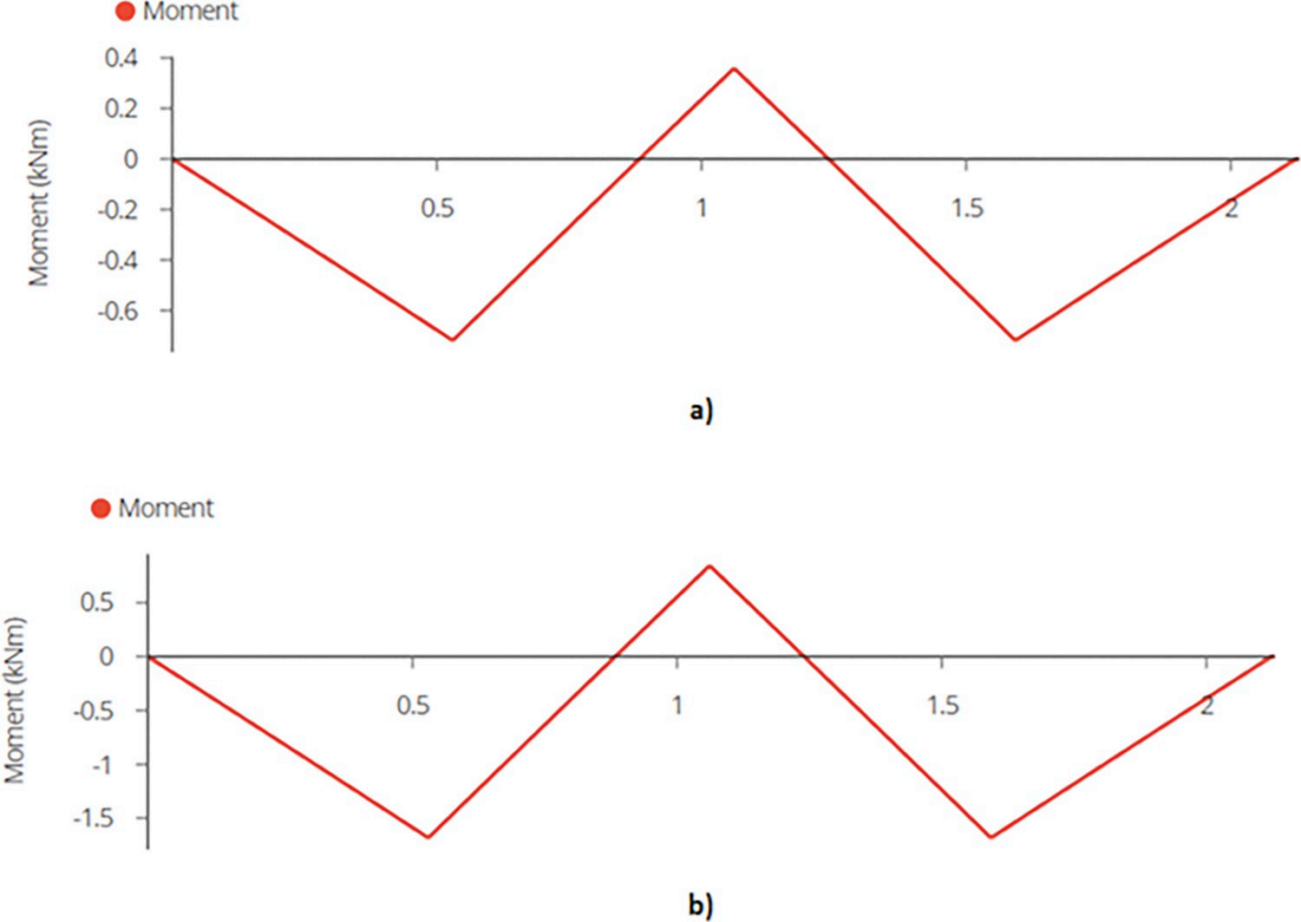
Bending Moment Diagram for Beams for a) 4-panel Structure and b) 4-panel Structure.

The maximum bending moment on the beam is 0.714 kN-m and 1.67kN-m. Fig 21A and 21B represent the bending moment diagram.

**Fig 22:**
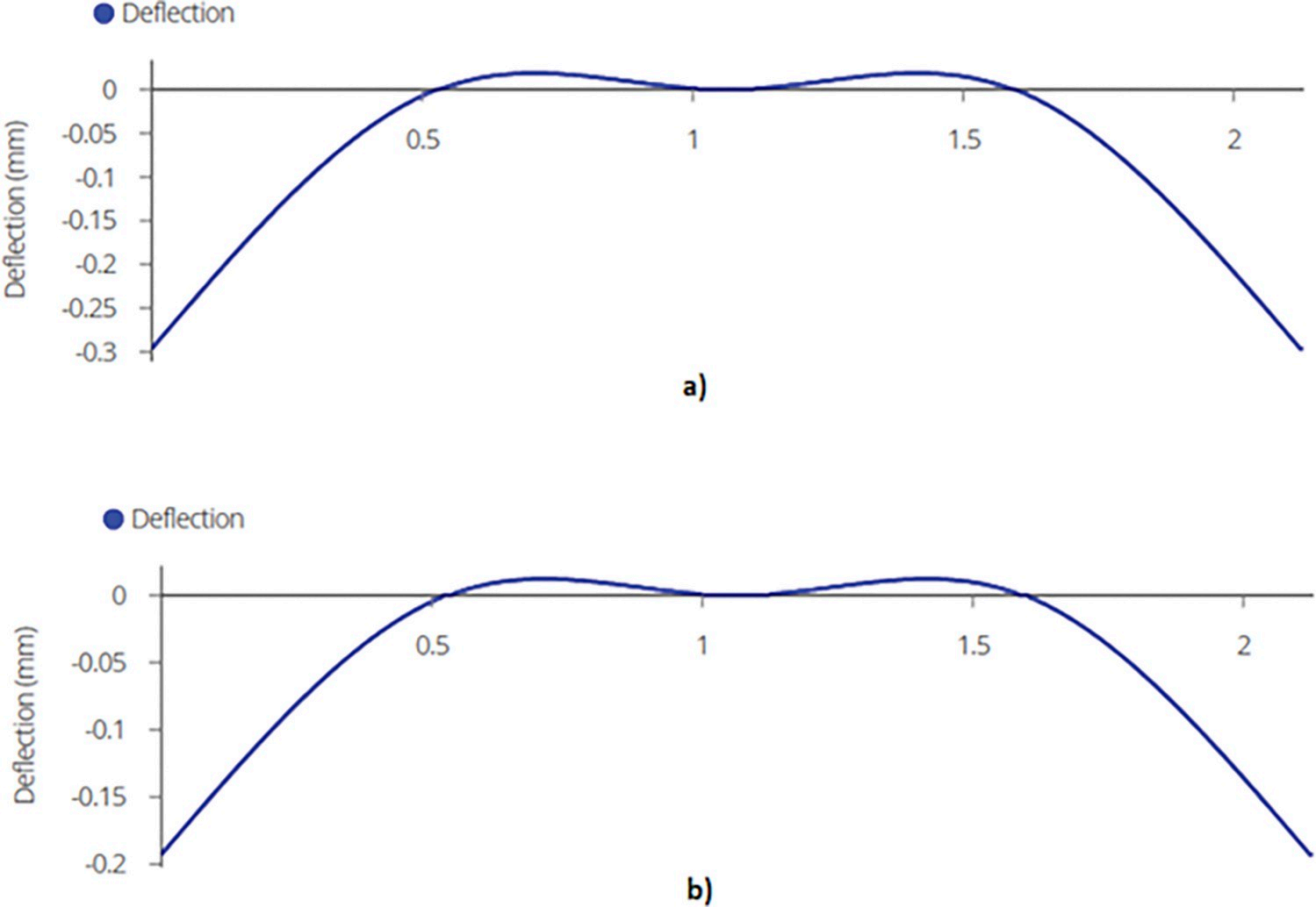
Deflection on Beams of a) a 920W Racking Design and b) a 1840W Racking Design.

Finally, the maximum deflection is determined to be 0.296mm and 0.193mm as shown in Fig 22A and 22B.

All the values are less than the resistance values of the members considered for the analysis, hence, they can be used to construct the racking structure. The structural analysis for the columns, however, must be performed.

The load from the beams is next transferred to the posts.

#### 4.3.4. Posts

**Fig 23:**
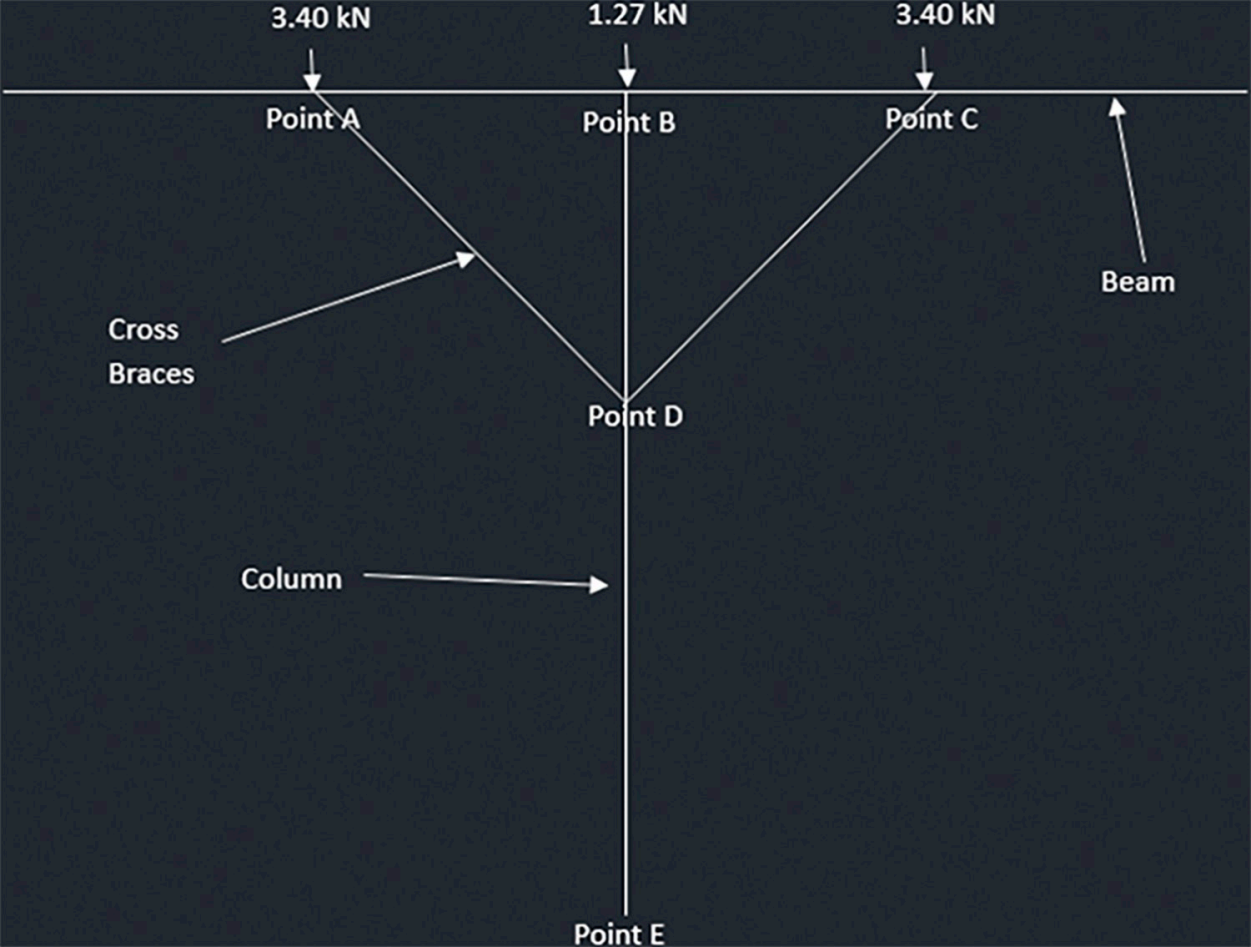
Truss analysis for 2-panel structure.

**Fig 24:**
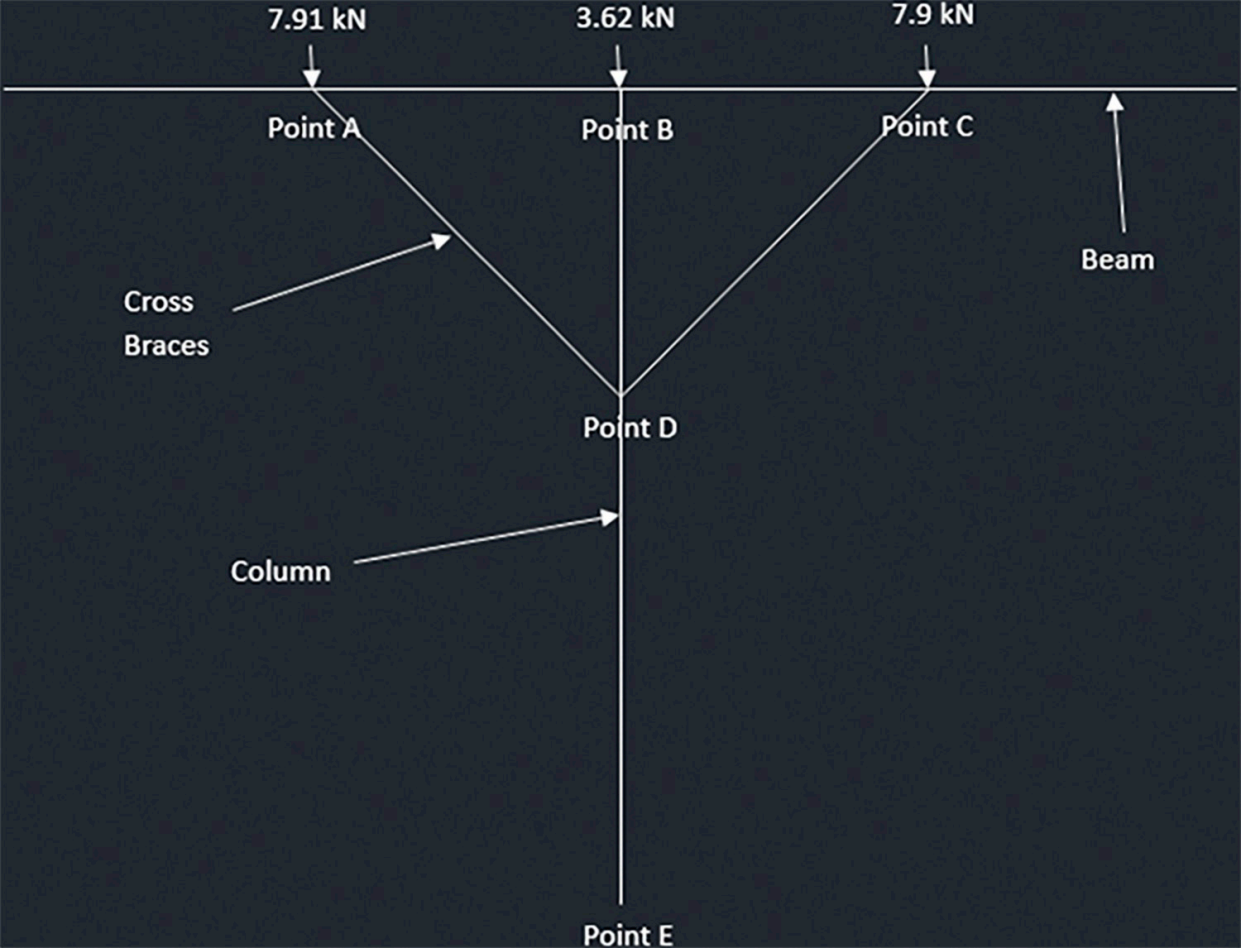
Truss analysis for 4-panel structure.

**Fig 25:**
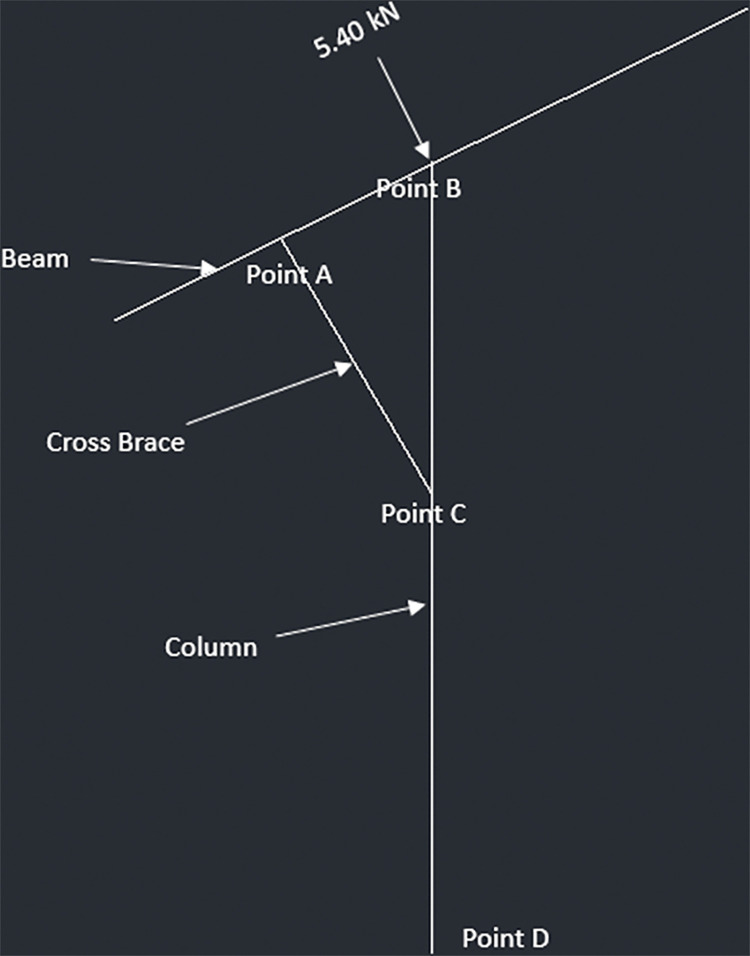
Truss analysis for sloped T-shaped racking configuration.

**Fig 26:**
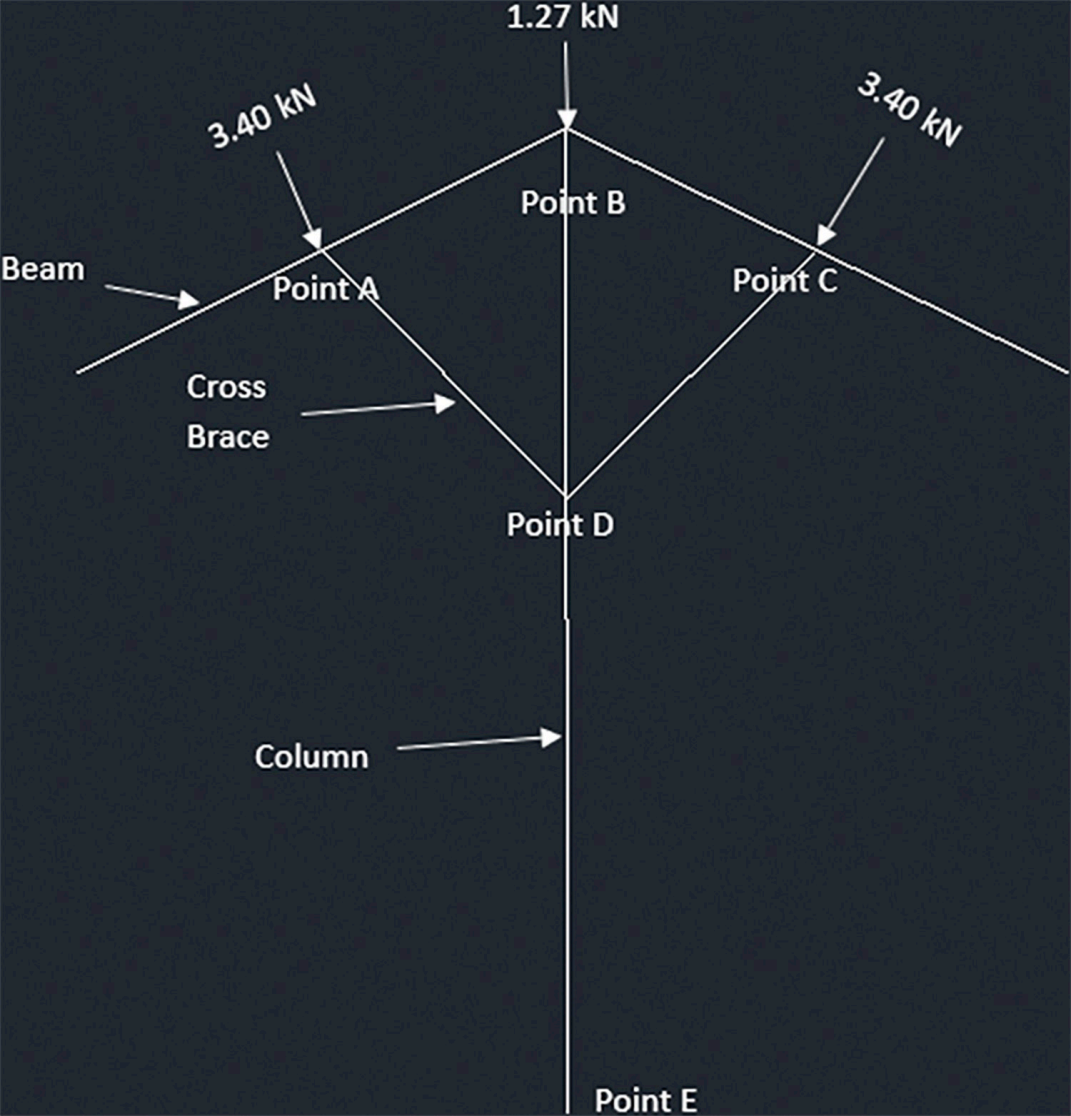
Truss analysis for inverse Y type racking design.

The allowable force comes out to be 223.43kN for 6x6 columns. The following diagrams (Figs 23–26) represent the truss made up of beam, cross braces and the column for T-shaped PV racking design with 2 panels, T-shaped racking design with 4 panel, sloped PV racking configuration and inverse Y racking configuration. The calculation for each truss type is shown in S4 Appendix. The truss analysis shows satisfactory results.

For the ground, if the applied pressure exceeds the allowable limit, two options can be considered. First, 150 mm of compacted clear stone gravel can be added to the bottom of the footing. Alternatively, the diameter of the footing can be increased.

Within the entire system, the load is transferred from one member to another through shear forces within the fasteners that form the connections. The shear resistance of a 1/2" carriage bolt holding the beams, complying with ASTM A307A, is approximately 23.8 kN. Similarly, the shear resistance of a 1/4" lag bolt holding the modules is 5.21 kN [20]. Both values exceed the demand of the systems and are therefore not critical to the design.

**Fig 27:**
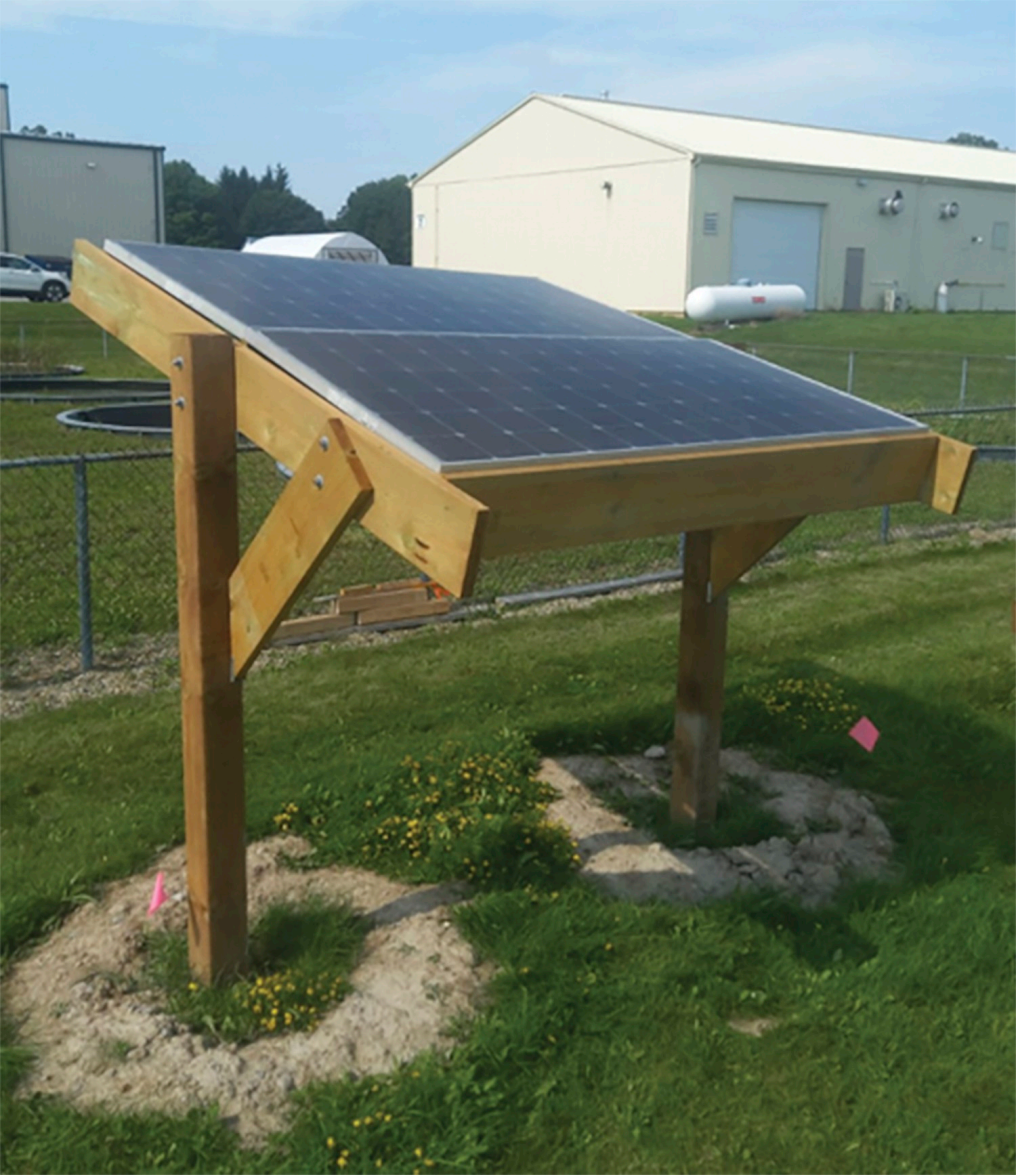
Sloped T-shaped PV racking.

Sloped T-shaped PV racking design was constructed following the instructions found in S5 Appendix (Fig 27).

The annual AC energy output and energy yield simulations for various tilt angles were also performed. For a 4-panel racking design, annual AC energy yield for the 1^st^ year is found out to be 1,967kWh with a 1069kWh/kWp of electrical output.

Similarly, analysis for the 2-panel racking design was also performed. For the 1^st^ year, annual energy yield for 920W racking design came out to be 998kWh while the energy output for 1 kW of installed solar panels was found out to be 1084kWh.

For inverse Y design, the 1^st^ year annual energy output came out to be 947 kWh for N-S orientation. A 1-kW installation with similar orientation will generate 1028.5 kWh in a year. E-W facing 920W rack will provide 947.5 kWh of energy output for the first year. A 1-kW system of similar orientation will generate 1029.5 kWh of electrical energy.

**Fig 28:**
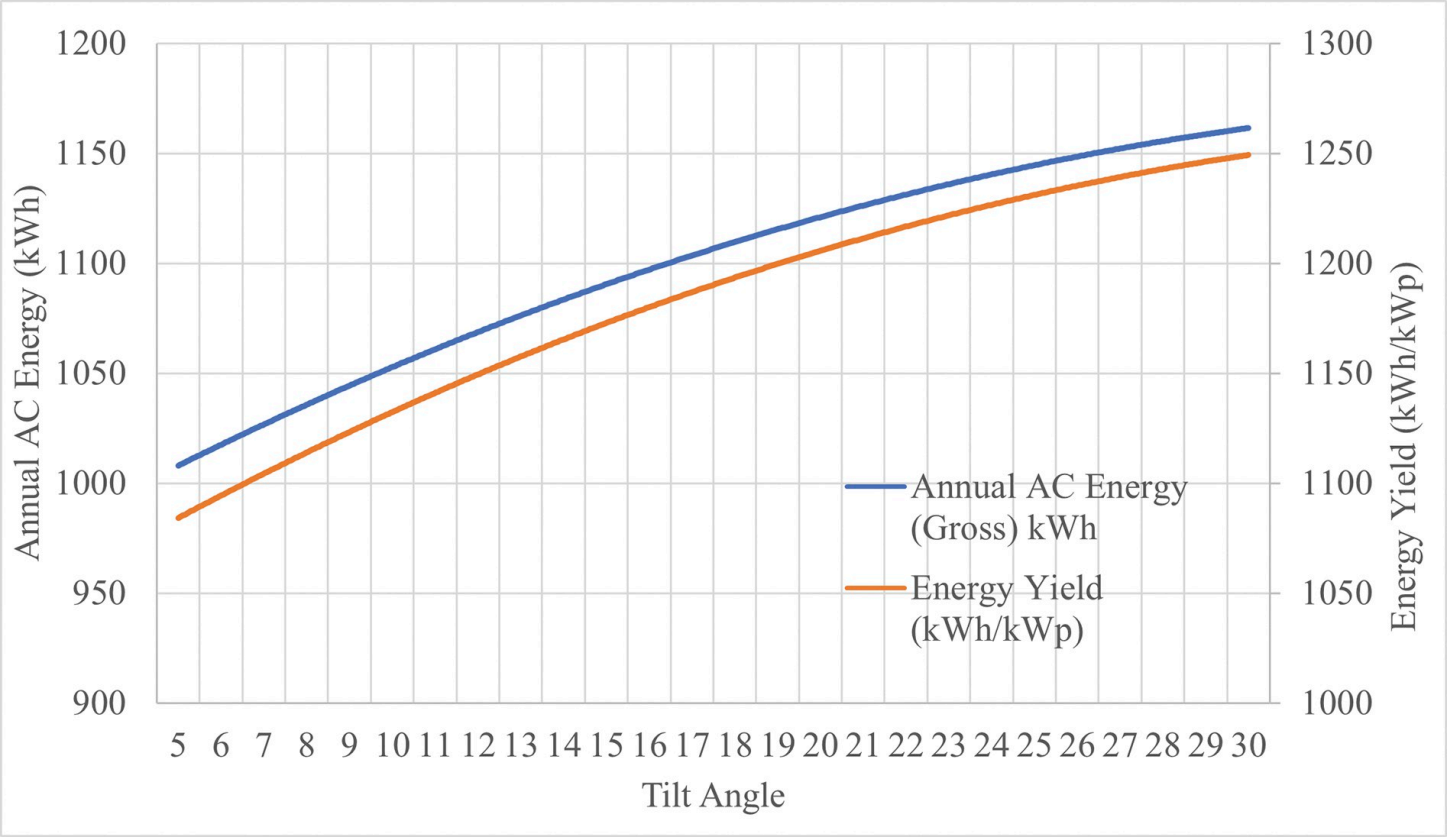
Impact of different tilt angles on energy output in Kelowna, BC.

Fig 28 shows the energy output and the corresponding tilt angles for the 920W 2-panel design from 0 to 30 degrees, the latter of which is the maximum tilt angle tolerated by a 6x6 wood column.

Although the main focus of the article is on the design and engineering evaluation of the trellis-based racks, economic analysis is also performed using the cost of the build materials. A financial snapshot provides the users with an overview of the commercial viability of the system. Using the current pricing of all the material inputs including lumber, nuts, bolts, screws, nail, concrete/cement, 3-D printed clips, washers, brackets etc., the total material cost of each racking design is ascertained. Compared to the other wooden designs suggested in the literature, the proposed structure is competitive as shown in Table 9.

**Table 9:** Cost comparison of different types of wooden PV racks.

Racking System	Cost (CAD)	Cost (CAD/Watt)
Fixed Racking Configuration [20]	426 (389)	0.35 (0.32)*
Variable Tilt Racking Configuration [27]	438 (406)	0.36 (0.34)*
Vertical Wood Racking Configuration [28]	371 (300)	0.15 (0.13)*
T-shaped Racking Configuration (2-panel Design)	397	0.43
T-shaped Racking Configuration (4-panel Design)	1155	0.63
Sloped Racking Configuration	372	**0.40**
Inverse Y Racking Configuration	427	0.46
Fixed Racking Configuration (Modified to 1.8m with 6x6 columns)	526	0.44
Variable Tilt Racking Configuration (Modified to 1.8m with 6x6 columns)	598	0.50

* Originally reported values in publications in brackets lower because of inflation

The fixed racking configuration and the variable tilt racking configuration have heights that are similar to the conventional PV mounting structures and do not go as high as 1.8m. In case the height of such structures was to be increased to the height of grape farm trellises, their cost becomes equal or even higher than the proposed T-shaped PV rack as can be seen in the modified configurations. Hence, the system offers the most cost effective agrivoltaics solution agrivoltaic systems that require trellises.

## 5. Discussion

This paper presents novel agrivoltaic PV racking designs for trellis-based crops. The main features of the proposed structures are simplicity and easy-to-build configuration. A few commercial entities such as Sun’Agri [77, 78], Ombrea [79, 80], Ibderdola [81], Huawei [82, 83], ANTAI and Mibet [84, 85] have come up with agrivoltaic racking solutions for trellis-based crops but the designs presented in this paper offer low-cost solutions for solar panel mounting on farmlands as well as flexibility to choose from, based on individual needs and requirements. Also, since they are made of wood, people might find them aesthetically pleasing, compared to structures made of metals, especially on agricultural lands. It may be worth mentioning that the racks will be economically viable in areas and regions where wood is low in cost and abundant. Thus, the finances of the racking configuration will vary based on the location where it is being built.

**Fig 29:**
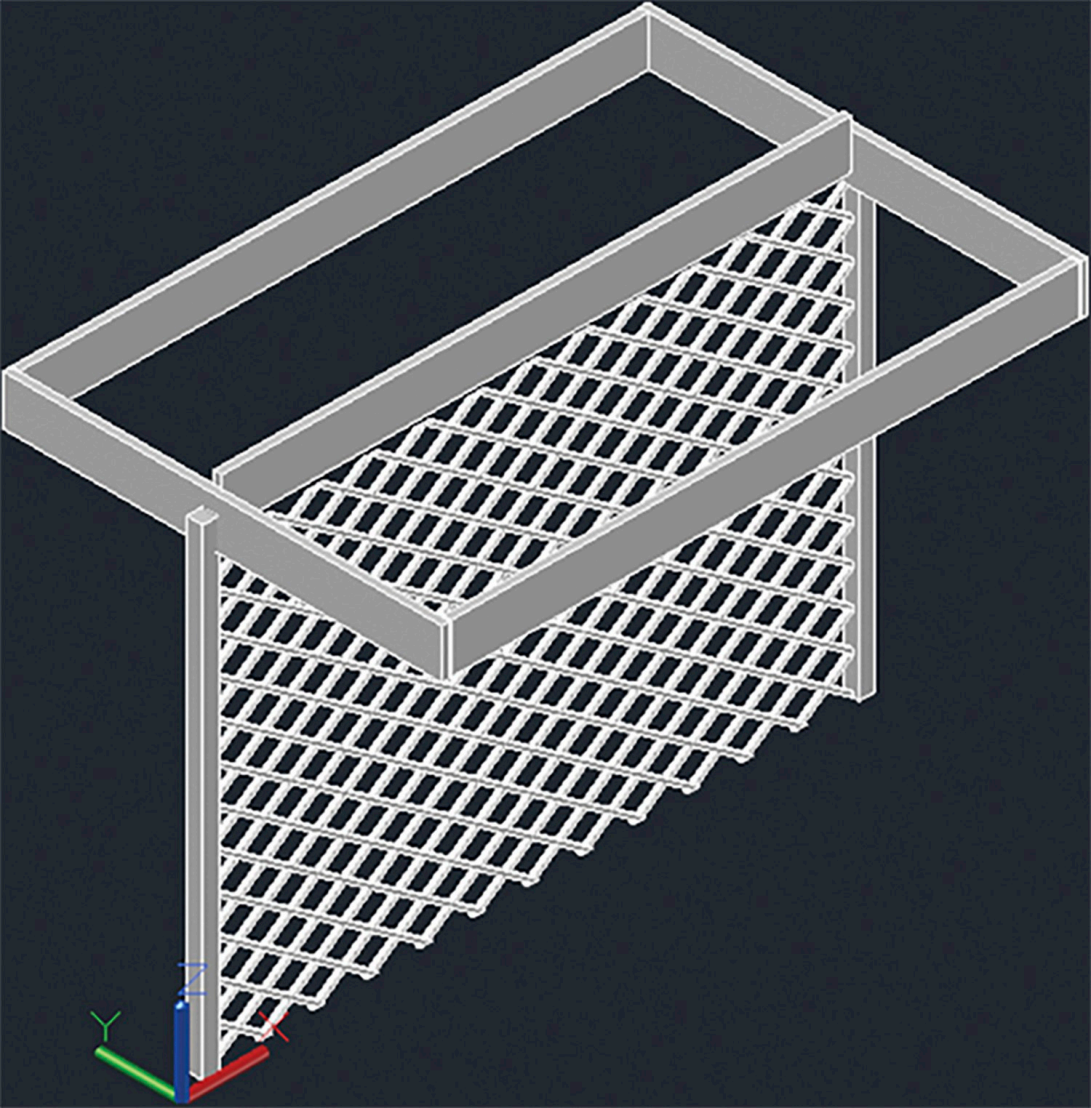
Trellis columns used as the posts for solar PV mounting.

The PV racks have been designed for plants that are grown using a trellis. The application of these structures may include crops such as cucumbers, grapes, kiwi, melons, peas, chayote, nasturtium, loofah, Malabar spinach, passion fruit, pole beans, pumpkins, strawberries, summer squash, tomatoes etc. The columns of the rack can be used to act as trellis for the crops or vice-versa i.e., the trellis posts could be used as columns for racks, provided they satisfy the structural integrity requirements as detailed in S2 Appendix. Fig 29 demonstrates such an application. Moreover, the racking can also be used to support and route piping for irrigation and fertigation purposes.

Irrespective of applied preservatives on lumber, the wooden structures which will be below the ground need to be appropriately rated for this, while the upper structures can have a ground contact but have a lower rating. A more stringent rating equates to a greater amount of preservatives being used on the lumber, which in turn translates into higher costs. If a treated wooden solar PV racking is installed as per the guidelines of the manufacturer, it can last up to 25-years [93], similar to the lifetime warranted for PV modules. Extra measures (such as hand treating exposed board cuts and drilled holes with a 2% copper naphthenate solution) need to be adopted if wooden structures are being built in areas prone to wood deterioration. The Forestry Chronicle provides a decay hazard map that highlights high decay hazards off the coast of British Columbia [105]. The American Wood Protection Association (AWPA) offers a decay hazard zone map indicating high decay hazards in California and the southern regions [106]. When treated wood reaches the end of its service life, it must be disposed of responsibly. In order to avoid landfilling of the treated wood, it can be diverted, and either be recycled or reused and repurposed for other services such as making wooden seats/chairs, and frames, etc. [90, 91]. Another option for disposing of waste wood is to use thermal processes such as low-temperature pyrolysis or high temperature gasification [92]. Thus the wood can converted into a number of valuable products. There is also a possibility of using naturally occurring decay-resistant wooden species such as white oak, white cedar or western red cedar, which have superior strength and decay resistance [107] as well as better mechanical properties [108]. It should be pointed out, however, these natural decay-resistant woods are generally expensive and might result in an economic disadvantage. Another limitation of the study is the variation in prices of wood. Based on the cost, the economic feasibility of these racks might vary in different parts of the world [87].

Furthermore, performing a comprehensive life cycle analysis (LCA) would ascertain whether this design, despite being constructed using sustainably harvested wood, is ecologically superior when compared to conventional metal-based PV racks. The evaluation should take into account all associated external costs and determine the system’s performance in different scenarios. In addition, practical experimentation of the racks and its implication of trellis-harvested foods should also be performed to confirm its viability in an agrivoltaic application. Moreover, future work can compare energy simulation results from SAM with real life measurements for the racking designs to determine if any small micro-climate impacts can be detected and quantified. On the short-term the weather had no impact on the PV racking, but, the mechanical stability of the racking design can be evaluated using strain measurements to ascertain any impacts of weather over the long term, which could lead to a more optimized design. Canada has vast acres of grape farms as discussed previously and with a 5m row spacing in between the panels for 920W rated racks, the installation potential of PV is approximately 10,219MW. Considering energy output of 1084 kWh for 1 kW-PV system installed in Kelowna, this provides renewable electrical energy potential of 11,077 GWh. To put into context, this means approximately 7,850,076 metric tons of CO_2_ equivalent could be reduced by employing agrivoltaics only to grape farms in Canada [109]. This is equivalent to burning 3988568.98 tons of coal, 1,746,880 gasoline driven vehicles annually and 2,716,289 tons of waste recycled instead of landfilled [109]. The results will be similar to carbon sequesteration offered by 9,361,354 acres of U.S. forest in one year if all the grape farms in Canada are employed with agrivoltaic systems [109].

The cost of a 2.1m x 0.8m complete trellis with side columns and base is approximately $260 [110]. With the racking structure in place, there will be no need to buy a complete trellis, instead only the lattice could be purchased which can be placed/installed in between the two 6x6 column. The cost of 2.4m x 1.2m lattice is approximately $27 [111]. This will result in savings of upto $233 for a single trellis/agrivoltaic rack. Considering the prices of our two panel racking designs i.e., $372 (sloped racking configuration), $397 (T-shaped)and $427 (inverse Y racking), and the savings involved, the net expenditure on these racks will be $139, $164 and $194. This translates into $0.15/W for sloped, $0.18/W for T-shaped and $0.21/W for inverse Y racking prices respectively.

## 6. Conclusions

This paper presents the first open source low-cost trellis-based agrivoltaic racking designs. In total, four different racking configurations are designed for 920W and 1,840W ratings. Three different topologies for 920W rated structure included simple T-shaped, sloped racking and inverse-Y configuration. An economic analysis was also carried out which shows that the the novel racks are cost efficient when compared with the traditional wooden PV mounting structures discussed in the literature previously. The design provides distinct advantages to crops including utilizing the columns of the racks to act as trellis supports and using the structure for irrigation/fertigation purposes. The results indicate that if these racking structures are employed on grape farms inside Canada, the country could benefit from more 10 GW of renewable electricity. This is more than twice the total installed solar energy in Canada and half of the total wind and solar installations combined [112]. In British Columbia, the total installed capacity of wind and solar is less than 1 GW [112]. Adopting agrivoltaics on grape farms using the racking structures discussed in the paper will increase the province’s share towards renewable energy generation, and will help meet the Canadian environmental and climate-related goals.

## Supporting information

S1 Appendix Design analysis assumptions. (DOCX)

S2 Appendix Load calculations. (DOCX)

S3 Appendix SAM input parameters. (DOCX)

S4 Appendix Truss analysis. (DOCX)

S5 Appendix Build instructions. (DOCX)
