# Supplementary material for: Solar photovoltaic wood racking mechanical design for trellis-based agrivoltaics
Source: PLoS One. 2023 Dec 1;18(12):e0294682. doi: 10.1371/journal.pone.0294682 (PMC10691708; doi:10.1371/journal.pone.0294682)
Supplement: S1 Appendix — (DOCX) [file pone.0294682.s001.docx]

**Appendix A. Design Analysis Assumptions**

Several assumptions are made here to streamline the calculations; however, these assumptions are conservative to ensure the structure withstands the most extreme field conditions.

- All loads apply perpendicular to the module face. This also ensures the joists experience the most severe flexural load, which is a conservative analysis.
- For simplicity, all members are assumed to be connected with pins and have no fixed end moments, considering that joist hangers and brackets allow for rotation.
- Assuming compliance with NBC 4.1.6, the wind load and snow load are evenly distributed across the module surface, as considerations for snow and wind accumulation apply primarily to large structures.
- The Heliene 144HC M6 module can withstand a front load of up to 5400 Pa and a rear load of up to 2400 Pa. Since the design loads will be significantly lower than these values, the modules possess adequate structural capacity.
