# Supplementary material for: Solar photovoltaic wood racking mechanical design for trellis-based agrivoltaics
Source: PLoS One. 2023 Dec 1;18(12):e0294682. doi: 10.1371/journal.pone.0294682 (PMC10691708; doi:10.1371/journal.pone.0294682)
Supplement: S2 Appendix — (DOCX) [file pone.0294682.s002.docx]

**Appendix B**. **Load Calculations**

**B1. Snow Loads**

The specified snow load as per National Building Code of Canada (NBC) is given by:

S = I_s_[S_s_(C_b_C_w_C_s_C_a_)+S_r_] [kPa] Eq. (B.1-1)

The importance factor (I_S_) for snow load is taken as 1.15 since the failure of PV racking design poses a risk to the life of people working underneath the structure. It should be noted that this is a conservative estimate as 1.00 could be used because the risk of people being beneath the racks in a storm is very low.

The 1-in-50-year ground snow load factor (S_S_) is dependent on the location where the racking structure is being built. Using Table C-2 in NBC for Kelowna, BC, a value of 1.70 is determined.

The basic roof snow load factor (C_b_) is considered 0.80 if l_c_ is less than or equal to the factor (70/C_w_^2^). Here C_w_ is the wind exposure factor and l_c_ is the characteristic length of the upper or lower roof. Value of l_c_ can be determined using the equation 2w - w^2^/l where w is the smaller plan dimension of the roof and l is the larger plan dimension of the roof. For the design, l_c_ is found out to be less than (70/C_w_^2^).

The wind exposure factor (C_w_) is taken as 0.75 considering our racking structure will be exposed to wind in all directions.

The slope factor (C_s_) is dependent on the tilt angle of the system. Since the slope is less than 15 degrees, C_s_ is considered 1.

The accumulation factor (C_a_) is taken as 1.00 for small single slope structures.

The 1-in-50-year associated rain load (Sr) is dependent on the location where the racking structure is constructed. Using Table C-2 of NBC for Kelowna, BC, a value of 0.1 is ascertained.

Table B.1 summarizes the factors used for calculating snow load:

Table B.1: Snow Load Factors

| **Factor** | **Value** |
| --- | --- |
| Importance Factor (I_s_) | 1.15 |
| Snow Load Factor (S_s_) | 1.70 |
| Basic Roof Snow Load Factor (C_b_) | 0.80 |
| Wind Exposure Factor (C_w_) | 0.75 |
| Slope Factor (C_s_) | 1.00 |
| Accumulation Factor (C_a_) | 1.00 |
| Associated Rain Load (S_r_) | 0.10 |

**B2. Wind Load**

The specified wind load is calculated using National Building Code of Canada (NBC) 2020. The wind load is a summation of both external wind pressure as well as internal wind pressure.

W = p + p_i_ [kPa] Eq. (B.2-1)

W is the total wind load in kPa

p is the specified external wind pressure in kPa

p_i_ is the specified internal wind pressure in kPa

The following equations govern the external and internal wind pressures:

p = I_w_qC_e_C_t_C_p_C_g_ [kPa] Eq. (B.2-2)

pi = I_w_qC_ei_C_t_C_gi_C_pi_ [kPa] Eq. (B.2-3)

The wind importance factor (I_w_) taken as 1.15 since the failure of PV racking design poses a risk to the life of people working underneath the structure.

The reference velocity pressure (q) is dependent on the location where the racking structure is being built. Using Table C-2 in NBC for Kelowna, BC, a value of 0.47 is determined.

The exposure factor (C_e_) is calculated using the expression (h/10)^0.2^ where h is considered 6m since the height of the structure is less than 20m as well as less than the smaller plan dimension. Value of C_e_ comes out to be 0.90.

The topographic factor (C_t_) is taken as 1.00.

The external pressure coefficient (Cp) and gust effect factor (Cg) are combined, and the value of the product is found out to be -1.30. Table 4.1.7.6. is used to determine the value.

The exposure factor for internal pressure (C_ei_) is the same as the exposure factor ‘Ce’ since wind can attack the inside of the system in the same way as the outside.

The internal gust effect factor (C_gi_) is taken as 2.00.

The internal pressure coefficient (C_pi_) is taken -0.70.

Table B.2 summarizes the factors used for calculating snow load:

Table B.2: Wind load Factors

| **Factor** | **Value** |
| --- | --- |
| Wind Importance Factor (I_w_) | 1.15 |
| Reference Velocity Pressure (q) | 0.47 |
| Exposure Factor (C_e_) | 0.90 |
| Topographic Factor (C_t_) | 1.00 |
| External Pressure Coefficient and Gust Effect Factor ‘Cp.Cg’ | 1.00 |
| Exposure Factor for Internal Pressure (C_ei_) | 0.90 |
| Internal Gust Effect Factor (C_gi_) | 2.00 |
| Internal Pressure Coefficient (C_pi_) | -0.70 |

**B3. Dead Load**

The structure's dead load ‘D’, which comprises the PV module weight and the wooden member weights, is the weight of the structure itself. The weight of brackets and fasteners is insignificant compared to the design load and can be considered negligible.

**B4. Load Combinations**

To account for the simplified assumptions made during the evaluation process, it is necessary to apply safety factors to the specified loads in order to minimize the likelihood of failure. These adjusted loads, known as factored loads, are then combined as principal loads and companion loads using the load combinations specified in Section 4.1.3.2 of NBC. The calculations should consider the combination of principal loads and companion loads that results in the highest net load. Principal loads are the essential loads that must be evaluated, while companion loads are included only if they act in the same direction as the principal loads. It is important to note that the design wind load acts in the negative direction, while the governing snow load is in the positive direction. Therefore, combining these loads would decrease the net load and is therefore not recommended.

**B5. Wooden Members Structural Capacity**

As discussed earlier, the materials properties of wood are variable due to the changes in moisture content and the presence of knots that alter the load path. The National Design Specification for Wood Construction (NDS) [80] provides reliable design values for a variety of wood types.

While these capacities have demonstrated reliability, it is essential to incorporate resistance factors into the capacities to accommodate unforeseen vulnerabilities and guarantee a design that is both safe and functional.

The load duration factor (C_D_) is taken as 1.15 considering typical design load, i.e., the governing load, as snow load.

The temperature factor (C_T_) is considered 1.00 since the racking structure is not expected to be exposed to a temperature of above 100^o^F.

The wet service factor (C_M_) is found out to be 1.00 for calculating factor f_b_, f_c_ and f_t_, 0.97 for factored f_v_ and 0.90 for factored E and E_min_

The beam stability factor (C_L_) is calculated as 0.76 for 2-panel design and 0.97 for 4-panel design according to the guidelines in Section 3.3.3. of National Design Specification [103].

The flat use factor (C_fu_) is taken as 1.20 for 2-panel design and 1.1 for 4-panel design as per table 4B of NDS Supplement [103] .

The incising factor (C_i_) is ascertained from table 4.3.8 of NDS and comes out to be 0.8 except for when determining factored E and E_min_ where its value is considered 0.95 [103].

The repetitive member factor (C_r_) is 1.00.

The size factor (C_F_) is 1.10 as per table 4A of NDS Supplement except for when determining factored f_c_ where its value is taken 1.00. For 4-panel design, the values are taken as 1.1 for f_b_ and 1.0 for f_c_ and f_t_.

The compression factor (C_P_) (calculated using the dimensions of the posts) is calculated in accordance with Section 3.7.1. of NDS and comes out to be 0.47for 6x6 members.

The National Design Specifications for Wood Construction provide the formulae for calculating the factored properties. The formulae are given below:

Factored bending stress = f_b_* = f_b_C_D_C_M_C_t_C_L_C_F_C_fu_C_i_C_r_ [MPa] Eq. (B.5-1)

Factored shear stress = f_v_*=f_v_C_D_C_M_C_t_C_i_ [MPa] Eq. (B.5-2)

Factored tensile stress = f_t_*=f_t_C_D_C_M_C_t_C_F_C_i_ [MPa] Eq. (B.5-3)

Factored compressive stress = f_c_*=f_c_C_D_C_M_C_t_C_F_C_i_C_P_ [MPa] Eq. (B.5-4)

Factored Elastic modulus = E*=EC_M_C_t_C_i_ [MPa] Eq. (B.5-5)

Factored E_min_ = E_min_*=E_min_C_M_C_t_C_i_C_T_ [MPa] Eq. (B.5-6)

Using the factored capacities calculated from above equations as well as the dimensional properties of the wooden members, the resistance values were finally calculated. The formulae for calculating the same are given below:

Resisting bending moment = M_r_ =(2f_b_*I/h) [kN-m] Eq. (B.5-7)

Resisting shear force = Vr =(f_v_*Ib/Q) [kN] Eq. (B.5-8)

Resisting tensile force = Tr =f_t_*A [kN] Eq. (B.5-9)

Resisting compressive force = Cr =f_c_*A [kN] Eq. (B.5-10)

For each member, certain conditions must be met to ensure structural integrity of the system and prevent its failure. These conditions include:

The resisting bending moment (M_r_) should be equal to or greater than the maximum applied bending moment (M_max_).

The resisting shear force (V_r_) should be equal to or greater than the maximum applied shear force (V_max_).

The resisting tensile force (T_r_) should be equal to or greater than the maximum applied tensile force (T_max_).

The resisting compressive force (C_r_) should be equal to or greater than the maximum applied compressive force (C_max_).

Additionally, the maximum deflection (D_max_) should not exceed the member length divided by 360, as specified in NBC 9.4.3.

**B6. Structural Analysis For T-shaped Racking**

The net load is uniformly distributed across the surface of the modules. According to the module supplier Heliene, the panels possess adequate capacity to support these loads. Subsequently, the load is transmitted from the panels to the joists. Each joist bears its own weight in the form of a uniformly distributed load, denoted as 'w'.

w= 1.25 (OW) [kN] Eq. (B.6-1)

OW is the own weight of the member. The load is multiplied by a factor of 1.25 since it is a dead load. In addition, to ascertain the uniformly distributed design load, the design load is multiplied with the tributary width for the joist under analysis. Total uniformly distributed load (UDL) is the sum of own weight and design load.

For the 2-panel design analysis, 2x10x8’ wooden member is considered while 6x6 column is considered for the posts. For 4-panel design, 4x10x16, 4x10x8, 2x10x8 wooden members are used with 6x6 posts. In case the applied values of bending moment, shear, compression, tension, and deflection are higher than the resistance values, a larger member should be considered for the analysis.

The maximum shear force or the reaction for the middle joist is ascertained using the following formula:

Maximum shear or reaction = Total UDL.L/2 [kN] Eq. (B.6-2)

where L is the length of the joist.

The maximum bending moment is determined using the following formula:

Maximum moment = Total UDL.L^2^/8 ----- [kN-m] Eq. (B.6-3)

Using the following equation, maximum deflection in the member is ascertained:

Maximum deflection = 5.Total UDL.L^4^/384EI ----- [mm] Eq. (B.6-4)

Analysis for the beam is next performed using Clearcals.

Subsequently, the buckling check for the posts is performed using the Euler Buckling equation given below:

C_buckling_ = π^2^EI_weak_/(Lk)^2^ [MPa] Eq. (B.6-5)

Where the effective length factor, k, is considered 2 for a column fixed at one and free on the opposite end.

Next, beam, cross braces and the column are considered to be a truss structure and a truss analysis is performed to check the forces acting on each of the members. The reaction forces from the beam are considered as the forces acting on the truss.
