# Supplementary material for: Solar photovoltaic wood racking mechanical design for trellis-based agrivoltaics
Source: PLoS One. 2023 Dec 1;18(12):e0294682. doi: 10.1371/journal.pone.0294682 (PMC10691708; doi:10.1371/journal.pone.0294682)
Supplement: S3 Appendix — (DOCX) [file pone.0294682.s003.docx]

**Appendix C. SAM Input Parameters**

| Parameters | 2-Panel Design | 4-Panel Design |
| --- | --- | --- |
|  |  |  |
| System Type | Detailed PV Model, Single Owner | Detailed PV Model, Single Owner |
| PV Module | Heliene 144HC-460 Bifacial | Heliene 144HC-460 Bifacial |
| Module Type | Mono Crystalline Silicon - Bifacial | Mono Crystalline Silicon - Bifacial |
| Number of Modules | 2 | 4 |
| Tilt Angle | From 5^o^ till 30^o^ | 5^o^ |
| Azimuth | 180^o^ | 180^o^ |
| DC Power Rating | 0.92 kWdc | 1.84 kWdc |
| DC to AC Ratio | 0.61 | 0.74 |
| Soiling Losses | 5% | 5% |
| DC Power Losses | 4.44% | 4.44% |
| AC Power Losses | 1% | 1% |
| PV Degradation Rate | 0.5% | 0.5% |
| Lifetime | 25 years | 25 years |
