# Supplementary material for: Solar photovoltaic wood racking mechanical design for trellis-based agrivoltaics
Source: PLoS One. 2023 Dec 1;18(12):e0294682. doi: 10.1371/journal.pone.0294682 (PMC10691708; doi:10.1371/journal.pone.0294682)
Supplement: S4 Appendix — (DOCX) [file pone.0294682.s004.docx]

**Appendix D. Truss Analysis**

**D1. *Calculations For 2-panel T-shaped Design:***

*Point A*

Using summation of forces as 0 across point A; the forces acting along the member F_AB_ and F_AD_ is determined.

ΣF_x_=0

F_AB_ + F_AD_cos(45) = 0 Eq. (D.1-1)

ΣF_y_=0

2.85 + F_AD_cos(45) = 0 Eq. (D.1-2)

F_AD_ = -4.80 kN

Using equation (A), F_AB_ = 3.40kN

*Point B*

ΣF_x_=0

F_AB_ – F_BC_ = 0 Eq. (D.1-3)

F_BC_ = 3.40 kN

ΣF_y_=0

F_BD_ + 1.27 = 0 Eq. (D.1-4)

F_BD_ = -1.27 kN

*Point D*

ΣF_x_=0

F_AD_cos(45) - F_CD_cos(45) = 0 Eq. (D.1-5)

F_CD_ = F_AD_ = -4.80 kN

ΣF_y_=0

F_AD_cos(45) + F_CD_cos(45) + F_BD_ + F_DE_ = 0 Eq. (D.1-6)

F_DE_= -8.06 kN

***D2. Calculations For 4-panel T-shaped Design:***

*Point A*

Using summation of forces as 0 across point A; the forces acting along the member F_AB_ and F_AD_ is determined.

ΣF_x_=0

F_AB_ + F_AD_cos(45) = 0 Eq. (D.2-1)

ΣF_y_=0

7.91 + F_AD_cos(45) = 0 Eq. (D.2-2)

F_AD_= -11.19 kN

Using equation (A), F_AB_= 7.91 kN

*Point B*

ΣF_x_=0

F_AB_ – F_BC_ = 0 Eq. (D.2-3)

F_BC_=7.91 kN

ΣF_y_=0

F_BD_ + 3.62 = 0 Eq. (D.2-4)

F_BD_ = -3.62 kN

*Point D*

ΣF_x_=0

F_AD_cos(45) - F_CD_cos(45) = 0 Eq. (D.2-5)

F_CD_ = F_AD_ = -11.19 kN

ΣF_y_=0

F_AD_cos(45) + F_CD_cos(45) + F_BD_ + F_DE_ = 0 Eq. (D.2-6)

F_DE_= -19.45 kN

***D3. Calculations For 2-panel sloped T-shaped Design:***

Before performing the truss analysis, the maximum tilt that can be achieved for the design was determined. Using the lead combination and the self-weight of the panel, a point load was calculated which was acting at an angle on the column. The load was broken into horizontal and vertical axes. The horizontal component would create an overturning moment while the vertical component would pass through the center of the column, hence, not contributing to the moment. Using the limiting bending moment of 6x6 column, horizontal component of the acting load and the length of the column, maximum tilt was determined which was found out to be 14.58^o^. So with a 6x6 column, any tilt below 14.58 ^o^ will be fine. To further increase the tilt, 8x8 column will have to be used which will allow inclination angle up to 35.16^o^. Truss analysis is performed for 14-degree tilt racking configuration.

Using summation of forces as 0 across point B; the forces acting along the member F_AB_ and F_BC_ is determined.

*Point B*

ΣF_y_=0

F_AB_sin (14) + F_BC_ + 5.40cos (14) = 0 Eq. (D.3-1)

ΣF_x_=0

F_AB_cos (14) + 5.40sin (14) = 0 Eq. (D.3-2)

F_AB_ = 1.34 kN

F_BC_= 5.36 kN

*Point A*

ΣF_x_=0

F_AC_cos(76) - F_AB_cos(15) = 0 Eq. (D.3-3)

F_AC_ = 5.37 kN

*Point A*

ΣF_y_=0

F_AC_cos(14) + F_BC_ + F_CD_ = 0 Eq. (D.3-4)

F_CD_= 10.54 kN

***D4. Calculations For 2-panel Inverse-Y Design:***

A similar methodology was adopted to perform the truss analysis for inverse Y racking design. The maximum tilt that could be achieved was determined by comparing the resisting bending moment with applied bending moment. Since the truss is identical, load acting on one half of the truss was considered. After determining the horizontal component, limiting bending stress and the distance from the column, the maximum inclination that could be achieved was found to be 30.41^o^.

Using summation of forces as 0 across point A; the forces acting along the member F_AB_ and F_AD_ are determined.

ΣF_y_=0

3.40cos(30) + F_AB_cos(60) – F_AD_cos(30) = 0 Eq. (D.4-1)

ΣF_x_=0

-3.40cos(60) + F_AB_cos(30) + F_AD_cos(60) = 0 Eq. (D.4-2)

F_AB_ = 0.00 kN

F_AD_ = 3.40 kN

*Point B*

ΣF_y_=0

1.27 - F_AB_cos(60) – F_BC_cos(60) - F_BD_ = 0 Eq. (D.4-3)

ΣF_x_=0

-F_AB_cos(30) + F_BC_cos(30) = 0 Eq. (D.4-4)

F_BC_ = 0.00 kN

F_BD_ = 1.27 kN

*Point D*

3.40 cos(30) + 1.27 + 3.40 cos(30) = F^­^_DE_  Eq. (D.4-5)

F_DE_ = 7.16 kN

Tension and compression values of all the members is within the resistance values. Also, the value for column is less the limiting value as determined from Euler Buckling equation. Ultimately, the load will be transmitted to the ground, where it will find its final support. The NBC's Table 9.4.4.1 presents the maximum permissible bearing pressures for various soil and rock types. In the most challenging scenario, soft clays have a maximum allowable bearing pressure of 75 kPa. To prevent overloading and excessive settling of the ground, the bearing pressure can be determined using the following equation:

Bearing Pressure = Post Compression/(π/4)(D_footing_)^2^ Eq. (D.4-6)
