# Supplementary material for: Solar photovoltaic wood racking mechanical design for trellis-based agrivoltaics
Source: PLoS One. 2023 Dec 1;18(12):e0294682. doi: 10.1371/journal.pone.0294682 (PMC10691708; doi:10.1371/journal.pone.0294682)
Supplement: S5 Appendix — (DOCX) [file pone.0294682.s005.docx]

**Appendix E. Build instructions**

1. Determine the slope/angle for the rack. Here 14^o^ is used as an example.
2. Draw slope lines on the columns (6x6) where the beam (2x10) will be bolted at an angle as shown in Figure E.1.


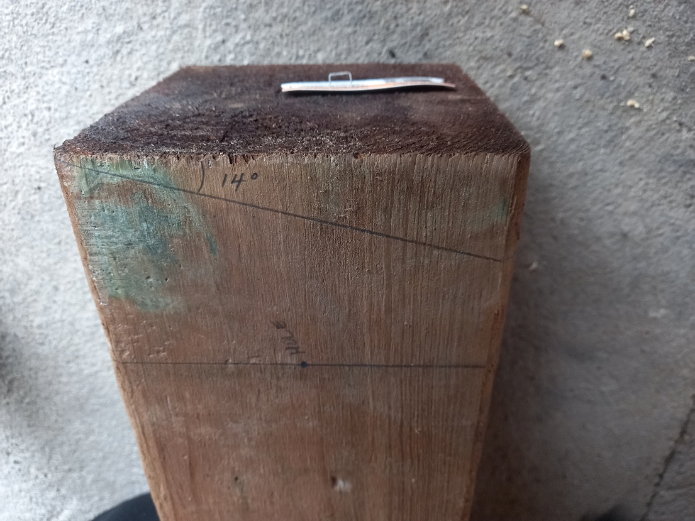


Fig E.1: Column with marking of slope

1. Find the center of the beam (2x10) and draw a line across the width.
2. From the center of the beam, draw a line at the tilt angle/slope for the rack i.e., 14^o^ (Fig. E.2).


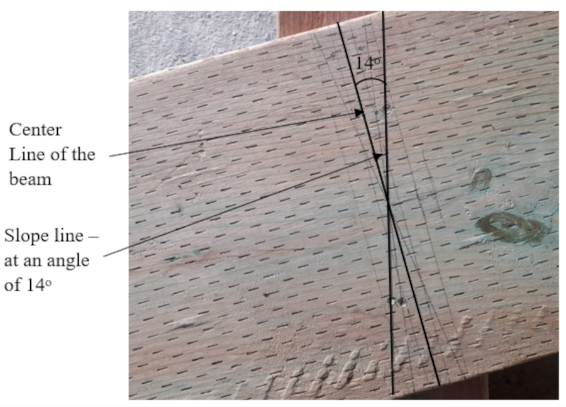


Fig E.2: Marking on the beam – centerline and tilt angle/slope

1. On the sloped line – mark location for the two holes at 1/4^th^ the distance of the total width of the beam from the top and 1/4^th^ the distance from the bottom (Fig. E.3).


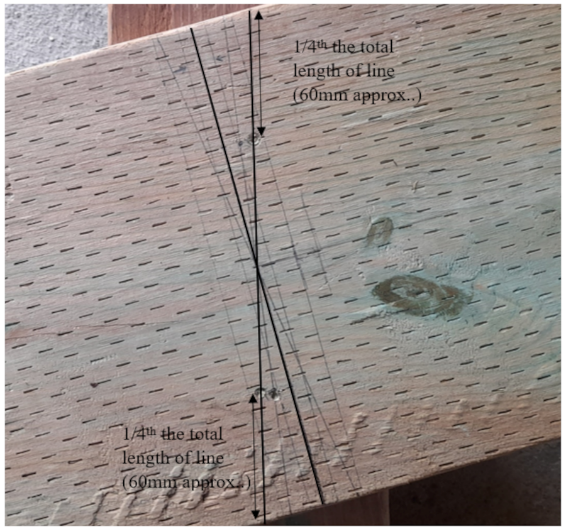


Fig E.3: Location of holes

1. Replicate the location of these hole on the 6x6 member. For this, draw a straight line starting from the top sloped line and ending at the bottom sloped line. The line should be drawn at the center of the column. Once the line is drawn, mark the location of the holes on the column. The distance of the holes on this vertical line will be the same as was on the beam’s sloped line (60mm approx.) (Fig. E.4).


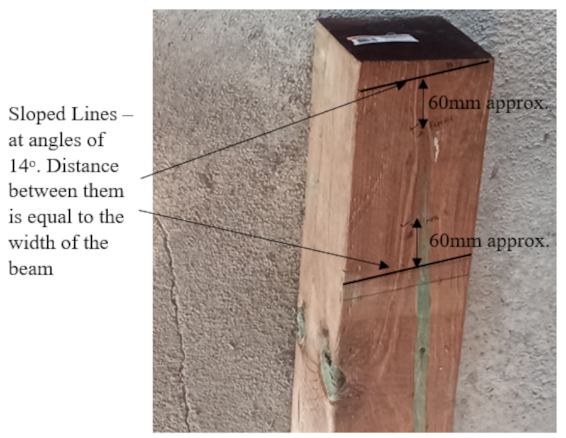


Fig E.4: Location of holes on the column

1. Drill two through holes (½”) through the beam and the column. For this, it is better to place the beam on the column on the sloped lines making the slope for the beam. Then use a small drill size to do the through holes in the beam such that an impression appears on the column. Then using the correct drill size, do through holes on both the column and the beam (See Fig. E.5, E.6 and E.7).


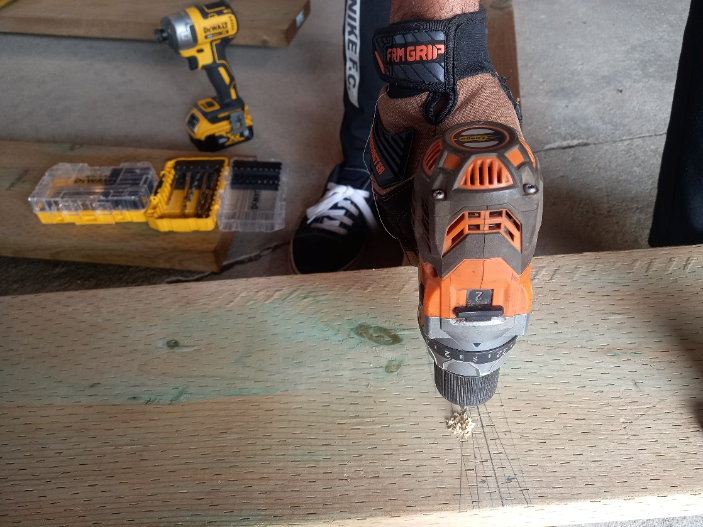


Fig E.5: Drilling holes in the beam


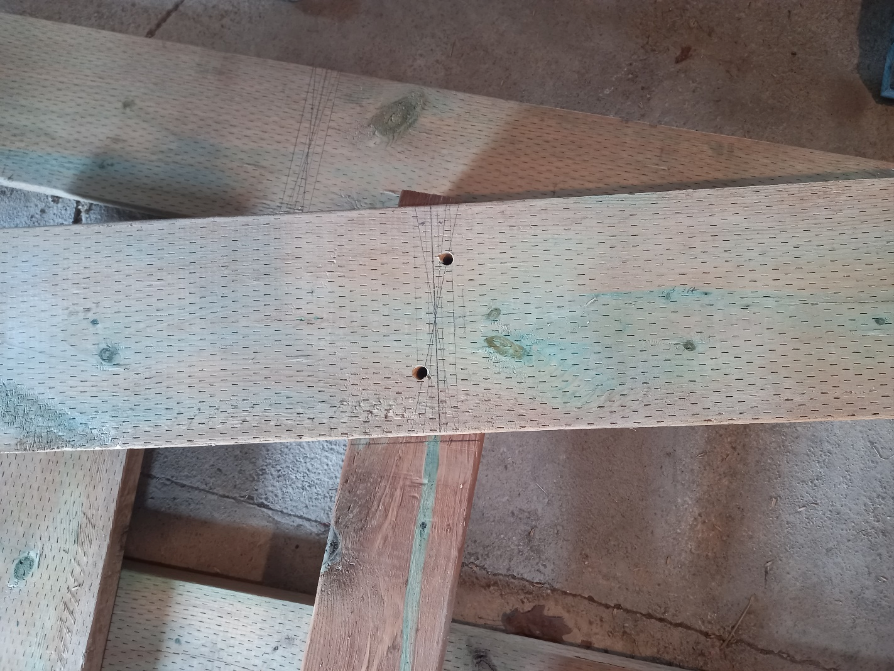


Fig E.6: Drilled holes on the beam (noted that the beam is placed on the column at the slope for which the rack is designed (14^o^).


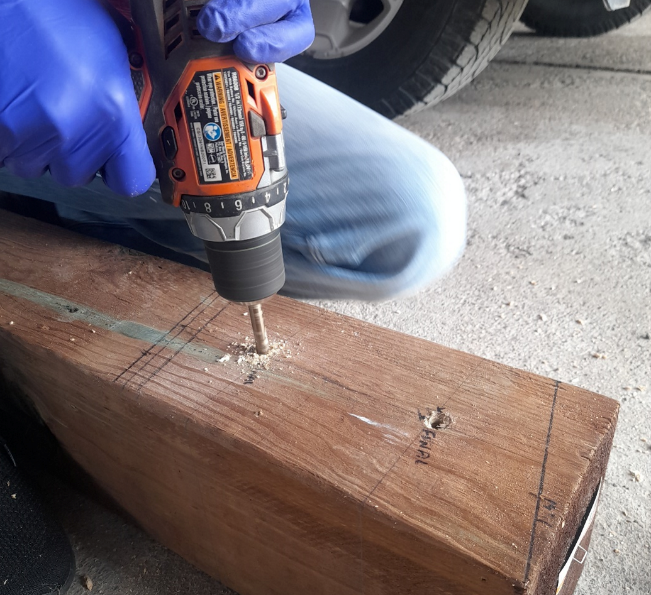


Fig E.7: Drilling holes in the column

1. Install the beams with the columns using 8” long carriage bolt along with washer, lock washer and nut (Fig. E.8, E.9) to get results (Fig. E.10).


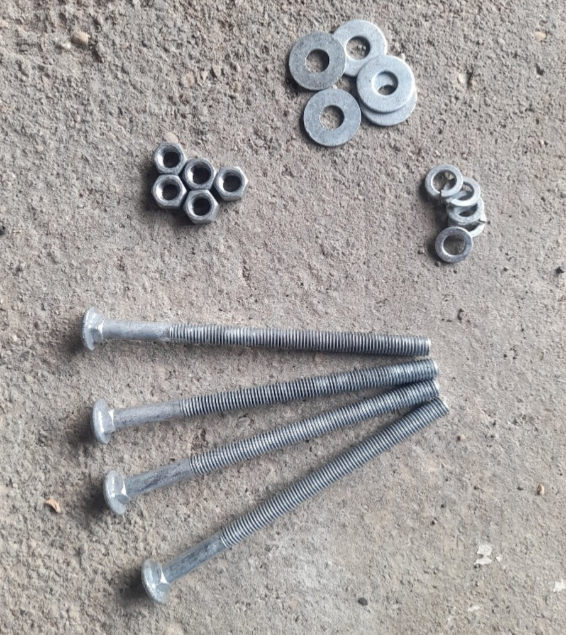


Fig E.8: Carriage bolts, washer, lock washer and nut


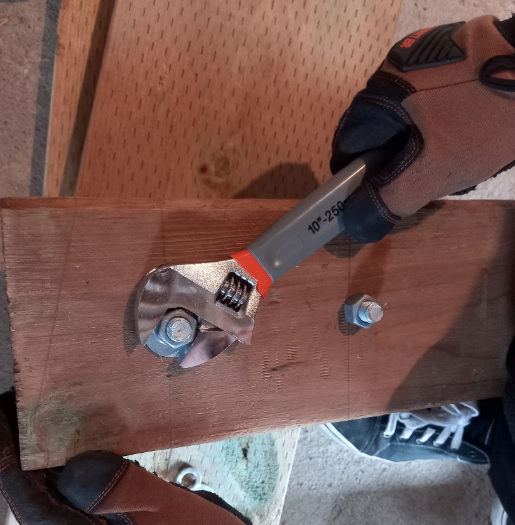


Fig E.9: Tightening the nut to couple beam with column


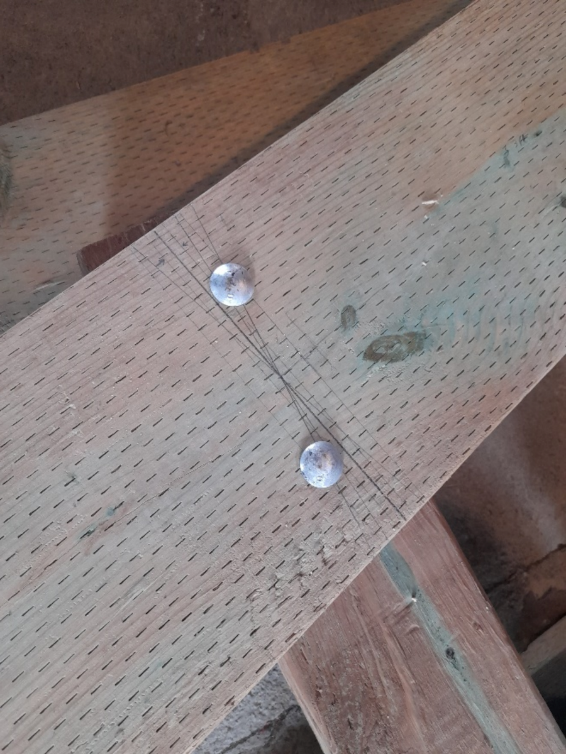


Fig E.10: Beam installed on the column

1. Repeat the same steps for the other column and beam. Note that they will be mirror image of the first one.
2. Using the dimensions in the drawing, find the location where the 2x4 fence brackets and and 2x6 facemounted joist hanger is to be installed. On the beams that area already attached to the columns, mark their location. Their location will largely depend on the size of panels which are being installed.
3. Install the facemounted joist hanger in the center of the beams. From the edge of facemounted joist hanger, install 2x4 fence brackets on either side of of the facemounted joist hanger at a distance of 1007mm (distance measured to the starting edge of fence bracket). Use 2-1/2” deck screws to screw both the fence brackets (Fig. E.11) and the joist hanger (Fig. E.12). (Note: The 2x4 fence brackets can also be installed on horizontal joist instead of the beams and then beams can slide into the fence brackets at the joists). See Fig. E.13.


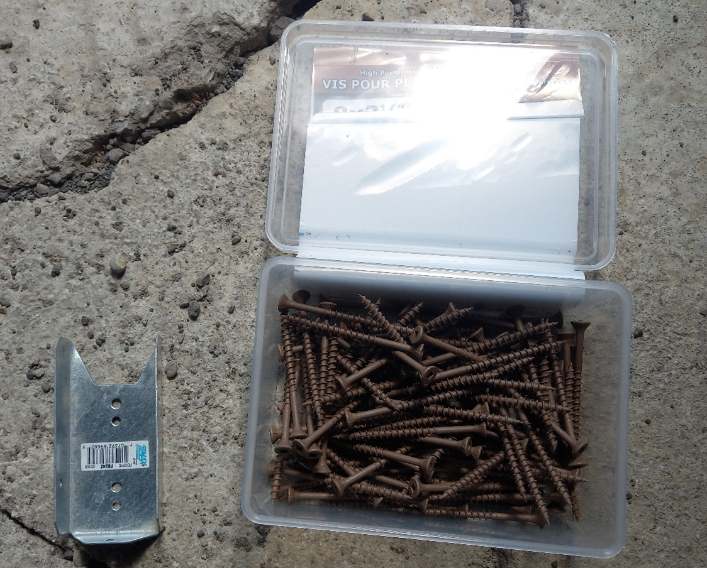


Fig E.11: 2x4 fence bracket and 2-1/2” deck screws


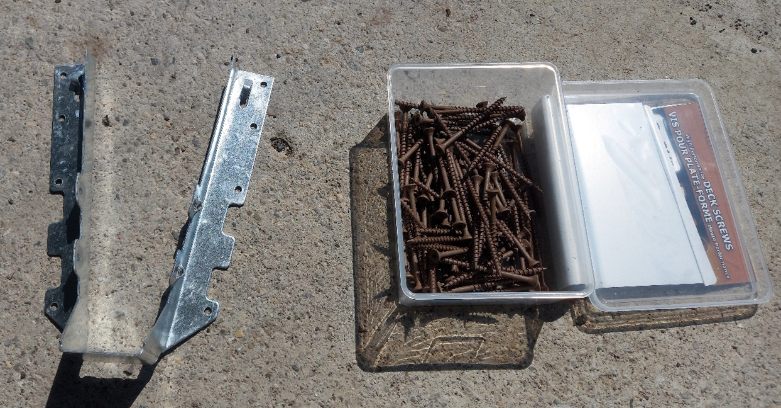


Fig E.12: 2x6 facemounted joist hanger and 2-1/2” deck screws


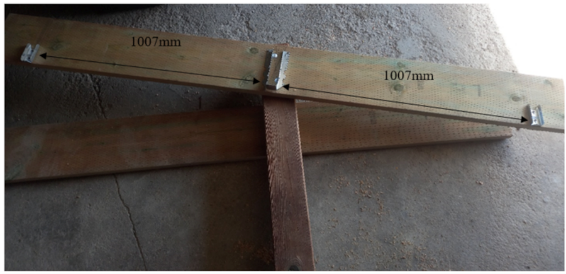


Fig E.13: Fence brackets and facemounted joist hanger installation

1. Next step to prepare the cross brace/diagonal to provide additional support to the beam (Fig. E.14). 2x10x8 member is cut into half so that we have two pieces of 2x10x4. One will be installed on each beam. The member is place at the mid-way between the joist hanger and fence bracket where it will be mounted using the carriage bolt.


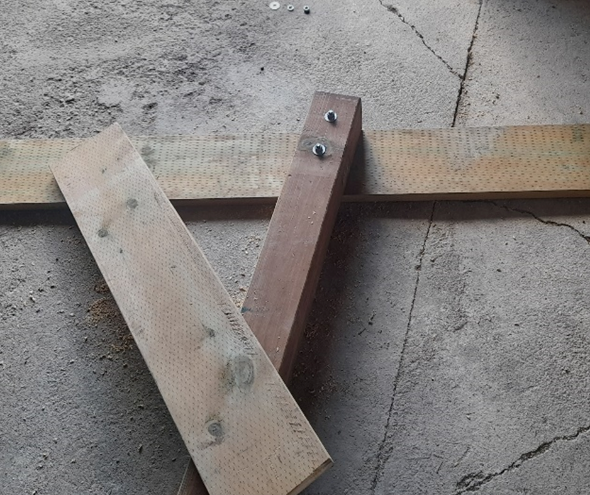


Fig E.14: Placing the cross brace to determine the cut location and the slope

1. Based on the angle of the diagonal, approx. 45^o^ for the prototype contruction, the diagonal member is cut. A mirror image of the diagonal for the second column is also prepared in a similar manner (Fig. E.15).


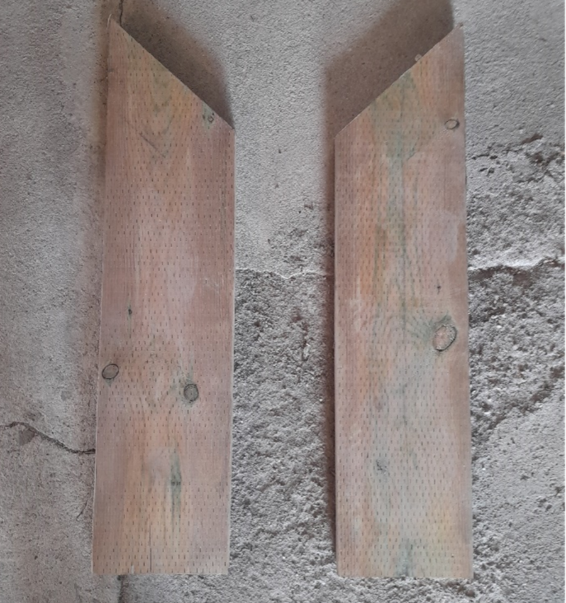


Fig E.15: Diagonal braces

1. The diagonals are placed on the beam flushed on the sides against the column Fig. E.16). A perpendicular line is drawn on the diagonal and location of the holes are marked keeping equal distance (1/4^th^ of the length of the line) from the top and bottom). Holes are drilled through the diagonal braces and the beam for their bolting.


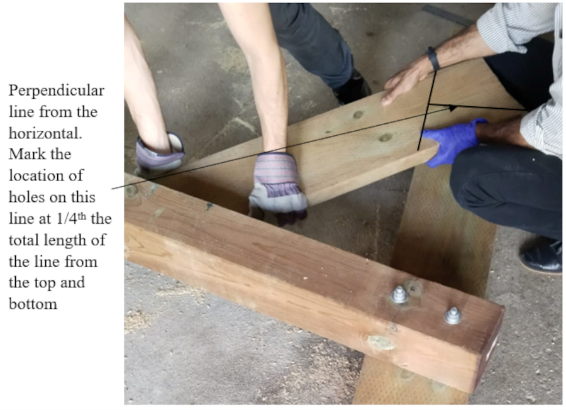


Fig E.16: Placing the diagonal across the beam

1. Next a 2x4 fence bracket is installed using 2-1/2” brown deck screws on the column considering the location where the cross brace will end up.
2. On the beam side, the diagonal are installed using carriage bolt, washer, lock washer and nut while nails are used at the fence bracket for attachment (Fig. E.17).


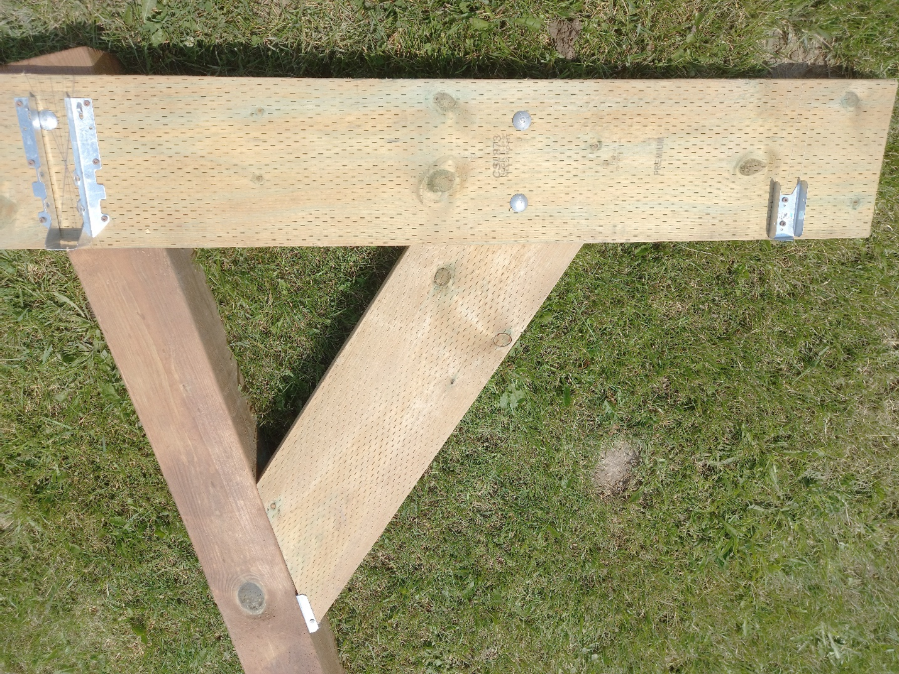


Fig E.17: Assembly of the diagonal with the column and beam

1. Two holes 1.2m in depth and 250mm in diameter are dug in the ground for the structure. The center-to-center distance between the holes in the ground is 2248mm. Place the columns in the ground.
2. Cut the three remaining 2x10x8 members to 2032mm in length. Install the center joist first by sliding it inside the facemounted joist hanger. Screw and nail it from the side. Similarly the other end joists are installed on the fence brackets using deck screws/nails. It is to be ensured that the columns are straight and in line with each other (Fig. E.18).
3. Once the structure is completed, pour in concrete/cement mixture inside the holes to fill it (an alternate way of the build could be to pour in concrete/cement mixture and let the columns set. Ensure that the two columns are in line, parallel and at correct distance so that the joists can slide in after the cement/concrete sets).


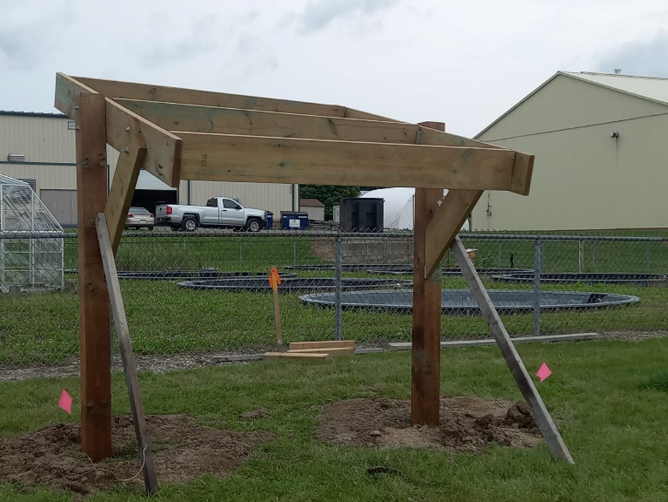


Fig E.18: Additional diagonal braces are installed to keep the structure upright/straight.

The other two racking configurations were constructed as well as shown in Figures E.19 (T-Shaped) and E.20 (inverse Y).


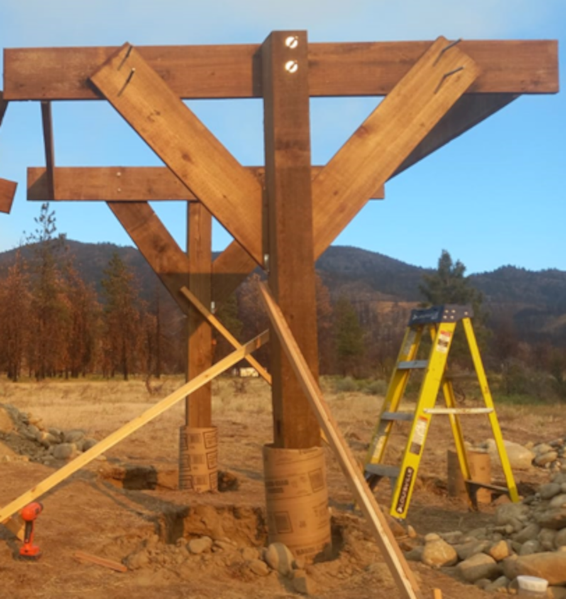


Fig E.19: Simple T-shaped racking configuration


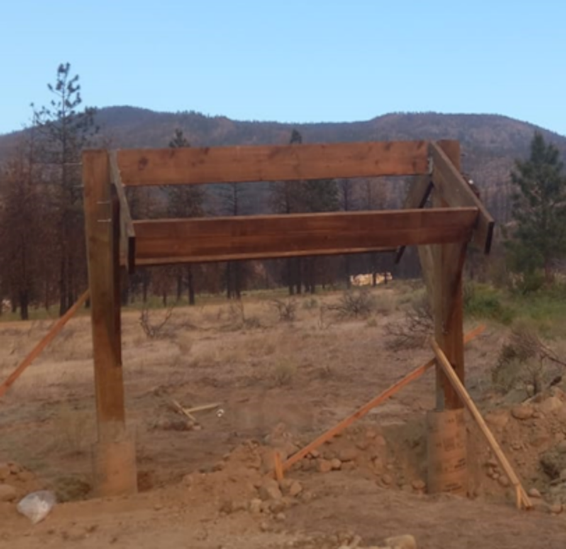


Fig E.20: Inverse Y racking configuration
